# Inhibiting ferroptosis enhances ex vivo expansion of human haematopoietic stem cells

**DOI:** 10.1038/s41556-025-01814-7

**Published:** 2025-11-18

**Authors:** Lucrezia della Volpe, Andrew J. Lee, Mateusz Antoszewski, Amy A. Deik, Ksenia R. Safina, Teng Gao, Chun-Jie Guo, Tianyi Ye, Peng Lyu, Jorge D. Martin-Rufino, Nicole Castano, Jonathan Good, Yaniris Molina-Aponte, Jiawei Zhao, Clary B. Clish, Peter van Galen, Vijay G. Sankaran

**Affiliations:** 1 Division of Hematology/Oncology, Boston Children’s Hospital and Department of Pediatric Oncology, Dana-Farber Cancer Institute, Harvard Medical School, Boston, MA USA; 2 Howard Hughes Medical Institute, Boston, MA USA; 3 Broad Institute of MIT and Harvard, Cambridge, MA USA; 4 Division of Hematology, Brigham and Women’s Hospital, Harvard Medical School, Boston, MA USA; 5 Department of Medicine, Harvard Medical School, Boston, MA USA; 6 Ludwig Center at Harvard, Harvard Medical School, Boston, MA USA; 7 Harvard Stem Cell Institute, Cambridge, MA USA; 8 Present Address: Faculty of Pharmaceutical Sciences, Shenzhen University of Advanced Technology, Chinese Academy of Sciences, Shenzhen, China

**Keywords:** Haematopoietic stem cells, Cell death, Bone marrow transplantation, Haematopoiesis

## Abstract

Improved ex vivo expansion of human haematopoietic stem cells (HSCs) would considerably advance transplantation and genome-engineered therapies, yet existing culture methods still allow substantial HSC loss. Here we show that this attrition is driven largely by ferroptosis, a metabolically regulated, iron-dependent cell-death pathway, and that it can be blocked to augment HSC expansion. Inhibiting ferroptosis with liproxstatin-1 or ferrostatin-1 markedly increases the expansion of cord blood and adult HSCs consistently across donors in both widely used serum-free cultures and recently reported chemically defined conditions. The expanded cells retain phenotypic and molecular stem cell identity and mediate improved durable, multilineage engraftment in xenotransplanted mice without genotoxicity or aberrant haematopoiesis. Mechanistically, ferroptosis blockade is accompanied by upregulated ribosome biogenesis and cholesterol synthesis, increasing levels of 7-dehydrocholesterol—a potent endogenous ferroptosis inhibitor that itself promotes HSC expansion. Crucially, this approach enhances yields of therapeutically genome-modified HSCs, paving a path for clinical applications.

## Abstract (web summary)

della Volpe et al. augment the ex vivo expansion potential of human haematopoietic stem cells (HSCs) by inhibiting ferroptosis with liproxstatin-1 or ferrostatin-1. Treated HSCs have enhanced in vivo repopulation capacity.

## Main

Tremendous advances in allogeneic haematopoietic transplantation from diverse cell sources and genome engineering of autologous haematopoietic stem cells (HSCs) have resulted in curative treatments for hundreds of thousands of patients^1,2^. However, the application of these approaches has been limited by inadequate cell doses in many instances^3^. The ability to maintain, expand and genetically modify HSCs ex vivo without compromising their functional properties for effective transplantation would enable therapies for a broader range of patients^4,5^. Even in successful trials, such as the recently reported and now approved use of CRISPR–Cas9 genome editing of the *BCL11A +*58 enhancer to cure sickle cell disease, all patients required between 3 and 18 days of apheresis after HSC mobilization to collect sufficient numbers of cells^6^. Such observations emphasize the limitations of current approaches to collect and manipulate human HSCs.

There have been many advances in culture conditions to maintain and expand human HSCs ex vivo. Initially, it was found that serum can promote HSC differentiation and serum-free conditions using albumin or polymers were identified that improved maintenance^7^. In addition, small molecules that prevent differentiation have been discovered, including the pyrimidoindole derivative, UM171, that alters the epigenetic state of haematopoietic cells^5,8^. Modulation of cytokine composition and concentration to maximally maintain HSCs has also shown value^9–11^. Recently, the development of chemically defined cytokine-free culture conditions has enabled the expansion of human HSCs over the course of weeks^12^. However, all of the advances in HSC expansion so far have focused on either preventing differentiation or maximizing quiescence. Given the complexity of the endogenous HSC niche^13–15^, it is likely that culture conditions fail to appropriately recapitulate all of the support present and thereby healthy HSCs might be lost as a result. Therefore, preventing the loss of HSCs could enable improved ex vivo HSC expansion. We have recently shown that in the setting of bone marrow (BM) failure, human HSCs display a unique vulnerability to loss by ferroptosis, a metabolically programmed form of cell death^16^. Here, we explored whether targeting this distinct vulnerability of human HSCs to ferroptosis might be exploited to improve expansion of human HSCs.

## Results

### Evaluating blockade of ferroptosis to enhance diverse culture systems

**Fig. 1:**
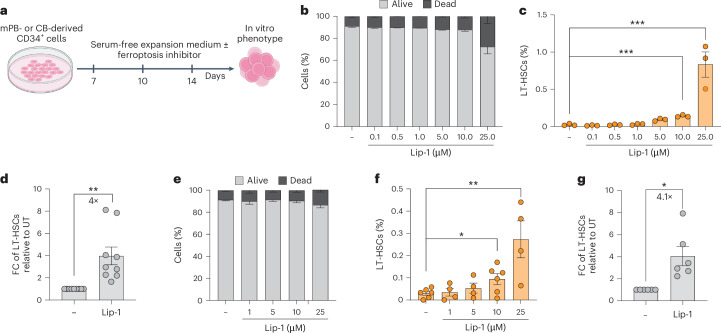
Ferroptosis prevention boosts the expansion of LT-HSCs regardless of the cell source. **a**, The experimental workflow: mPB- or CB-derived HSPCs were cultured for up to 14 days in a serum-free expansion medium in the presence or absence of ferroptosis inhibitors (Lip-1, Fer-1, sodium selenite or β-ME). **b**, Cell viability analysis of mPB HSPCs on day 14 was conducted using flow cytometry. The Apotracker probe allowed the detection of dead cells (*n* = 9, 3, 3, 3, 3, 9, 4). **c**, The percentage of LT-HSCs in mPB cells at day 14. The LT-HSC population was defined as CD34^+^CD45RA^−^CD133^+^CD90^+^EPCR^+^ITGA3^+^ (*n* = 3 per condition). Student’s *t*-test (*P* *=* 0.0006 and 0.0089). **d**, The fold change (FC) of mPB LT-HSCs at day 14 was calculated as the ratio of Lip-1 group % LT-HSCs/untreated group % LT-HSCs (*n* = 9 per condition). UT, untreated. One-sample *t*-test (*P* *=* 0.0059). **e**, A cell viability analysis of CB-HSP cells on day 14 was conducted using flow cytometry. The Apotracker probe allowed the detection of dead cells (*n* = 6, 4, 4, 6, 4). **f**, The percentage of CB LT-HSCs (CD34^+^CD45RA^−^CD133^+^CD90^+^EPCR^+^ITGA^+^) in CB cells at day 14 (*n* = 6, 4, 4, 6 and 4). Student’s *t*-test (*P* *=* 0.0361 and 0.0065). **g**, The fold change of CB LT-HSCs at day 14 was calculated as the ratio of Lip-1 group % LT-HSCs/untreated group % LT-HSCs (*n* = 6 per condition). One-sample *t*-test (*P* = 0.0312). Each dot represents an independent donor unless otherwise indicated and all the data are presented as mean ± s.e.m. Unless otherwise specified, a two-sided statistical test was used. *P < 0.05, **P < 0.01, ***P < 0.001 and ****P < 0.0001. Panel **a** created with BioRender.com. Source data

**Extended Data Fig. 1:**
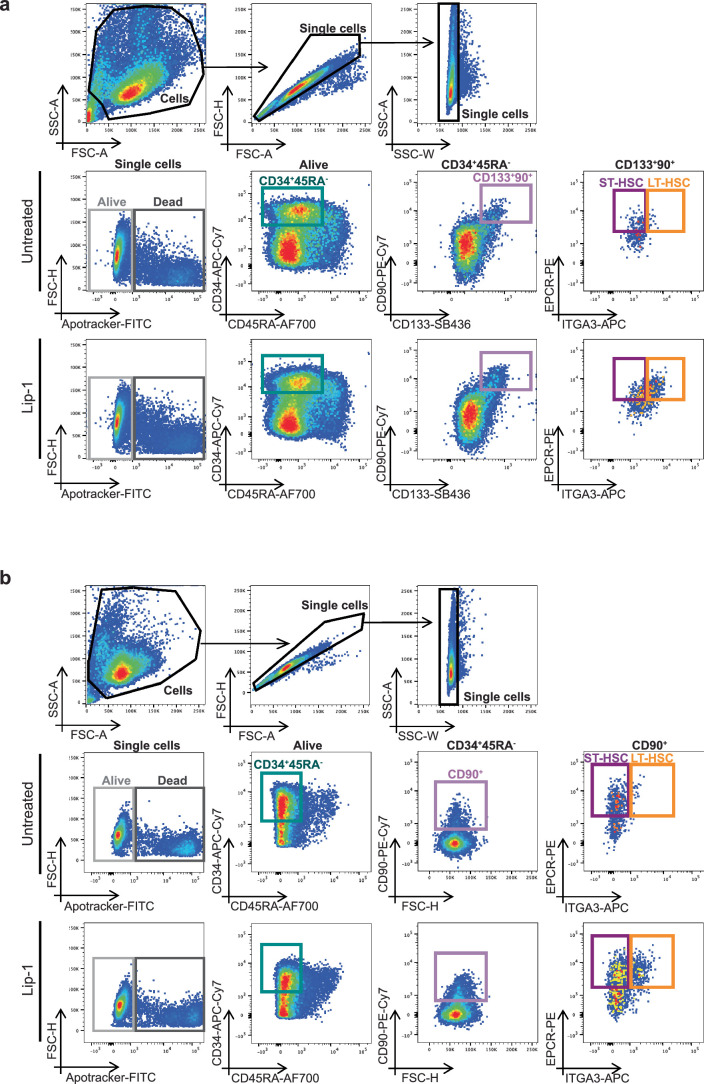
Phenotypic characterization of *ex vivo* expanded HSPCs. **a**, Representative plots and gating strategy for subset composition of HSPCs cultured according to the standard protocol. Firstly, we eliminated dead cells (Apotracker^+^) from the subsequent analysis, then, gating on live cells (Apotracker^−^), CD34^+^CD45RA^−^ cells were identified. Within this gate, we further gated on CD133^+^CD90^+^ (primitive HSPCs) and discriminated between EPCR^+^ITGA3^−^ (ST-HSC) and EPCR^+^ITGA3^+^ (LT-HSC) subsets. **b**, Representative plots and gating strategy for subset composition of HSPCs cultured according to the chemical defined cytokine-free protocol. Firstly, we eliminated dead cells (Apotracker^+^) from the subsequent analysis, then, gating on live cells (Apotracker^−^), CD34^+^CD45RA^−^ cells were identified. Within this gate, we further gated on CD90^+^ (primitive HSPCs) and discriminate between EPCR^+^ITGA3^−^ (ST-HSC) and EPCR^+^ITGA3^+^ (LT-HSC) subsets.

**Extended Data Fig. 2:**
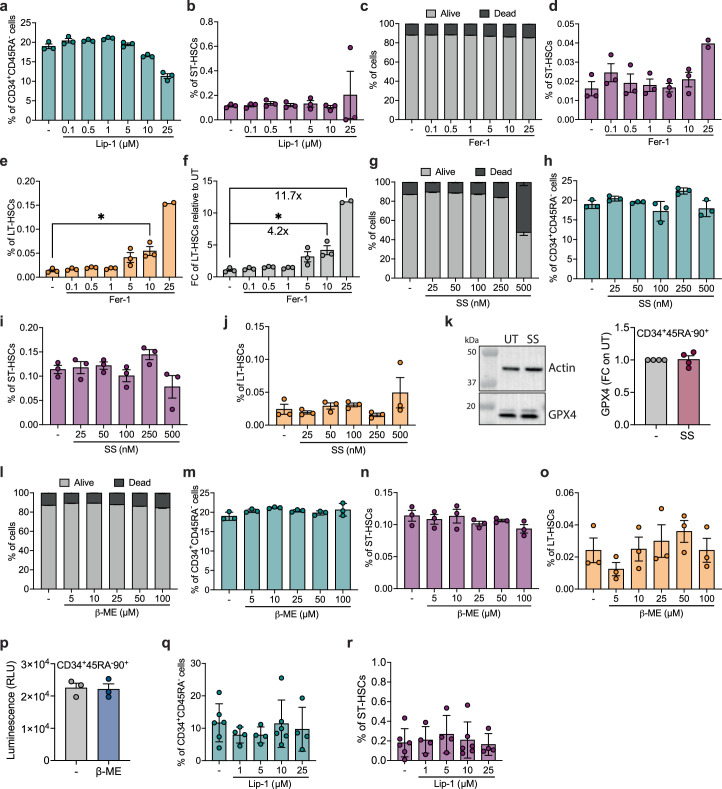
LT-HSC expansion is uniquely driven by RTAs, not by other metabolic inputs. **a, b**, Percentage of (**a**) CD34^+^CD45RA^−^ and (**b**) ST-HSC (CD34^+^CD45RA^−^CD133^+^CD90^+^EPCR^+^) in mPB cells at day 14 (n = 3 per condition). **c**, Cell viability analysis of mPB-HSPCs on day 14 was conducted using flow cytometry. The Apotracker probe allowed the detection of dead cells (n = 3 per condition). **d-e**, Percentage of (**d**) ST-HSCs (CD34^+^CD45RA^−^CD133^+^CD90^+^EPCR^+^), and (**e**) LT-HSCs (CD34^+^CD45RA^−^CD133^+^CD90^+^EPCR^+^ITGA3^+^) in mPB cells at day 14 (n = 3,3,3,3,3,3,2). Kruskal-Wallis test (p-values: 0.0427; 0.0165). **f**, Fold change of LT-HSCs at day 14 was calculated as the ratio of Fer-1 group %LT-HSCs / untreated group %LT-HSCs (n = 3,3,3,3,3,3,2). One sample t-test (p-values: 0.0464; 0.008). **g**, Cell viability analysis of mPB-HSPCs on day 14 was conducted using flow cytometry. The Apotracker probe allowed the detection of dead cells (n = 3 per condition). **h-j**, Percentage of (**h**) CD34^+^CD45RA^−^, (**i**) ST-HSCs (CD34^+^CD45RA^−^CD133^+^CD90^+^EPCR^+^), and (**j**) LT-HSCs (CD34^+^CD45RA^−^CD133^+^CD90^+^EPCR^+^ITGA3^+^) in mPB cells at day 14 (n = 3 per condition). **k**, Western blot analysis of GPX4 in CD34^+^45RA^−^90^+^ cells sorted on day 4 of culture in the presence of 250 nM SS (n = 4 per condition). Actin was used as a loading control, and fold change relative to control is reported. **l**, Cell viability analysis of mPB-HSPCs on day 14 was conducted using flow cytometry. The Apotracker probe allowed the detection of dead cells (n = 3 per condition). **m-o**, Percentage of (**m**) CD34^+^CD45RA^−^, (**n**) ST-HSCs (CD34^+^CD45RA^−^CD133^+^CD90^+^EPCR^+^), and (**o**) LT-HSCs (CD34^+^CD45RA^−^CD133^+^CD90^+^EPCR^+^ITGA3^+^) in mPB cells at day 14 (n = 3 per condition). **p**, GSH levels were measured in CD34⁺CD45RA⁻90⁺ cells immediately after sorting. Cells were collected after 4 days of expansion in medium supplemented with 100 µM b-ME (n = 3 per condition). **q, r**, Percentage of (**q**) CD34^+^CD45RA^−^ and (**r**) ST-HSCs (CD34^+^CD45RA^−^CD133^+^CD90^+^EPCR^+^) in CB cells at day 14 (n = 6,4,4,6,4). Each dot represents an independent donor unless otherwise indicated and all the data are presented as Mean ± SEM. Unless otherwise specified, a two-sided statistical test was used. *p < 0.05; **p < 0.01; ***p < 0.001; ****p < 0.0001. Source data

Although a number of inhibitors of ferroptosis have been identified, non-specific effects and undesirable properties limit the utility of many of these molecules. Radical trapping antioxidants such as ferrostatin-1 (Fer-1) or liproxstatin-1 (Lip-1) have been identified through high-throughput screens to potently prevent lipid peroxidation and ferroptosis^17–19^. Our prior studies had demonstrated the utility of both Lip-1 and Fer-1 in preventing ferroptosis in human HSCs^16^, and we therefore sought to apply such molecules in the context of existing human HSC ex vivo expansion approaches. We initially supplemented standard serum-free cultures used for human adult (mobilized peripheral blood, mPB) HSC maintenance with varying doses of Lip-1 and examined HSC content in the cultures using the phenotypic marker combination of CD34^+^CD45RA^−^CD90^+^CD133^+^EPCR^+^ that are known to enrich for bona fide human HSCs, even after ex vivo culture^20–23^ (Fig. 1a). This marker combination can be further stratified to separate long-term (LT) from short-term (ST) reconstituting HSCs based on ITGA3 surface expression on the former (Extended Data Fig. 1a). We found that Lip-1 did not cause cell toxicity, except at the highest dose of 25 µM (Fig. 1b). No major differences in the percentages of haematopoietic stem and progenitor cells (HSPCs) (CD34^+^CD45RA^−^ cells) or ST-HSCs compared with untreated cells were noted (Extended Data Fig. 2a,b). Importantly, there was dose-dependent preservation of LT-HSCs, with a ~4-fold increase in LT-HSCs at the 10 µM Lip-1 dose compared with controls after 2 weeks of culture (Fig. 1c,d). Notably, similar expansion was achieved when we supplemented the same serum-free culture medium with Fer-1 (Extended Data Fig. 2c–f).

While radical-trapping antioxidants such as Lip-1 and Fer-1 are potent inhibitors of ferroptosis, we wondered whether other metabolic inputs into this process could be manipulated to optimize the ex vivo culture of HSCs. The glutathione peroxidase GPX4 is a key seleno-enzyme involved in ferroptosis. We attempted to maximize GPX4 activity^24,25^ by optimizing selenium concentrations in cells with sodium selenite (Na_2_SeO_3_) supplementation, as has been shown previously in other cell contexts^24,26,27^. However, we observed no improvement in HSC maintenance nor any change in GPX4 protein levels, suggesting that selenium concentrations and GPX4 levels were already optimal in HSCs (Extended Data Fig. 2g–k). GPX4 utilizes reduced glutathione (GSH) to detoxify lipid peroxides and cystine is known to be rate limiting for the production of GSH. We added different concentrations of β-mercaptoethanol (β-ME), which serves as a cystine donor to promote GSH biosynthesis^28,29^, but once again observed no improvement in human HSC maintenance ex vivo (Extended Data Fig. 2l–o). Importantly, β-ME supplementation did not increase GSH levels in human HSCs, demonstrating that GSH availability is also not limiting in our culture conditions to maximally support GPX4 activity (Extended Data Fig. 2p). Therefore, these findings show that radical-trapping antioxidants that inhibit ferroptosis are uniquely able to prevent HSC loss in culture, and other specific metabolic inputs—selenium and GSH levels—do not appear to be limiting in cultured human HSCs.

Having shown improved ex vivo HSC maintenance and expansion in standard serum-free cultures with cells derived from adult sources, we examined whether this was also the case for HSCs obtained from cord blood (CB). CB-HSCs are frequently used for clinical haematopoietic transplantation and cell numbers obtained from CB units are often limited^3^. Akin to the results with adult human HSCs, we observed a ~4-fold expansion of CB-derived LT-HSCs after 2 weeks of culture with 10 µM Lip-1, without signs of toxicity or impacts on other subpopulations, indicating that blocking ferroptosis could broadly enable HSC expansion across a variety of cell sources (Fig. 1e–g and Extended Data Fig. 2q,r).

**Fig. 2:**
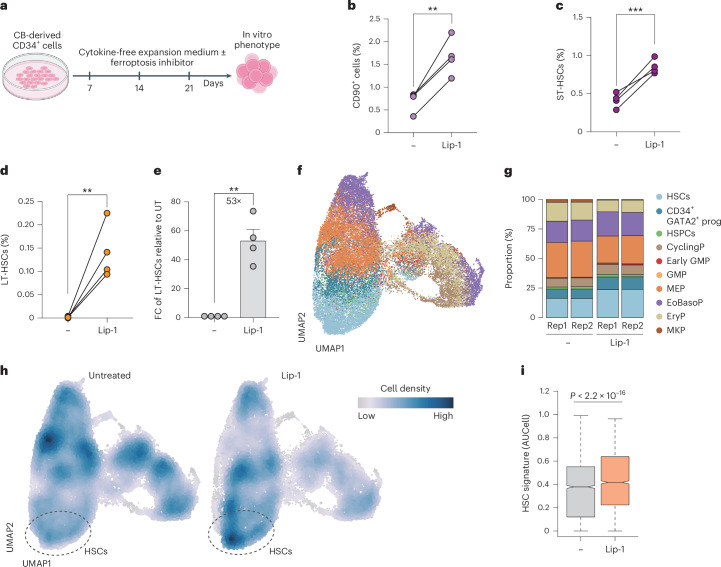
Ferroptosis inhibition enhances HSC expansion and enriches molecularly defined HSCs in chemically defined cultures. **a**, The experimental workflow: CB-derived HSPCs were cultured for up to 3 weeks in a cytokine-free expansion medium in the presence or absence of ferroptosis inhibitors (Lip-1 or Fer-1). **b**–**d**, The percentage of primitive HSPCs (defined as CD34^+^CD45RA^−^CD90^+^) (**b**), ST-HSCs (CD34^+^CD45RA^−^CD90^+^EPCR^+^) (**c**) and LT-HSC (CD34^+^CD45RA^−^CD90^+^EPCR^+^ITGA3^+^) (**d**) in CB cells at day 21 (*n* = 4 per condition). Student’s *t*-test (*P* = 0.0069 (**b**), 0.0007 (**c**) and 0.0035 (**d**)). **e**, The fold change of LT-HSCs at day 21 was calculated as the ratio of Lip-1 group % LT-HSCs/untreated group % LT-HSCs (*n* = 4 per condition). One-sample *t*-test (*P* = 0.0072). **f**, UMAP of 29,096 scRNA-seq cells sorted for primitive HSPCs (CD34^+^CD45RA^−^CD90^+^) based on the annotated cell population, comprising two conditions with or without Lip-1 treatment for 10 days (*n* = 2 replicates (Rep) per condition). **g**, A stacked bar plot showing the proportion of cell types assigned to individual samples (*n* = 2 per condition). EoBasoP, eosinophils–basophils progenitors; EryP, erythroid progenitors; GMP, granulocyte–macrophage progenitors; MEP, megakaryocyte–erythroid progenitors. **h**, UMAPs illustrating the cell state density between Lip1-treated and untreated control cells. The dotted grey eclipse indicates the HSC compartment. **i**, A box plot of *z*-score normalized HSC signature expression of all the cells of the untreated and Lip-1 groups (*n* = 2 per condition). The significance of differences in the two conditions was calculated based on a two-sided Wilcoxon rank-sum test. Each dot represents an independent donor unless otherwise indicated and all the data are presented as mean ± s.e.m. Unless otherwise specified, a two-sided statistical test was used. *P < 0.05, **P < 0.01, ***P < 0.001 and ****P < 0.0001. Panel **a** created with BioRender.com. Source data

**Extended Data Fig. 3:**
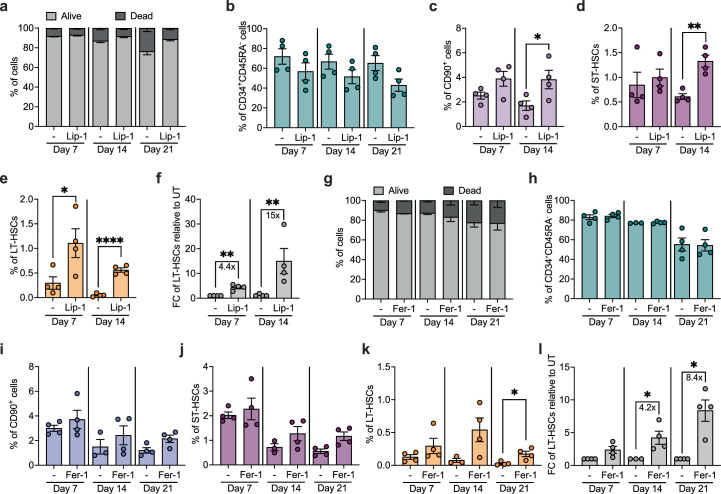
LT-HSC expansion-mediated by Lip-1 and Fer-1 in cytokine-free culture conditions. **a**, Cell viability analysis of CB-HSPCs at indicated time points was conducted using flow cytometry. The Apotracker probe allowed the detection of dead cells (n = 4 per condition). **b-e**, Percentage of (**b**) CD34^+^CD45RA^−^, (**c**) primitive HSPCs (CD34^+^CD45RA^−^CD90^+^), (**d**) ST-HSCs (CD34^+^CD45RA^−^CD133^+^CD90^+^EPCR^+^), and (**e**) LT-HSCs (CD34^+^CD45RA^−^CD133^+^CD90^+^EPCR^+^ITGA3^+^) in CB cells at indicated time points (n = 4 per condition). Student’s t-test (p-values: (c) 0.0145; (d) 0.0019; (e) 0.044; 0.0001). **f**, Fold change of LT-HSCs at day 14 was calculated as the ratio of Lip-1 group %LT-HSCs / untreated group %LT-HSCs (n = 4 per condition). Student’s t-test (p-values: 0.00108; 0.0359). **g**, Cell viability analysis of CB-HSPCs cultured in the presence of 5 µM Fer-1 was conducted at indicated time points using flow cytometry. The Apotracker probe allowed the detection of dead cells (n = 4,4,3,4,4,4). **h-k**, Percentage of (**h**) CD34^+^CD45RA^−^, (**i**) primitive HSPCs (CD34^+^CD45RA^−^CD90^+^), (**j**) ST-HSCs (CD34^+^CD45RA^−^CD133^+^CD90^+^EPCR^+^), and (**k**) LT-HSCs (CD34^+^CD45RA^−^CD133^+^CD90^+^EPCR^+^ITGA3^+^) in CB cells at indicated time points (n = 4,4,3,4,4,4). Student’s t-test (p-value: (k) 0.015). **l**, Fold change of LT-HSCs at day 14 was calculated as the ratio of Fer-1 group %LT-HSCs / untreated group %LT-HSCs (n = 4,4,3,4,4,4). One sample t-test (p-values: 0.0489; 0.0203). Each dot represents an independent donor unless otherwise indicated and all the data are presented as Mean ± SEM. Unless otherwise specified, a two-sided statistical test was used. *p < 0.05; **p < 0.01; ***p < 0.001; ****p < 0.0001. Source data

While serum-free culture approaches are commonly applied in clinical gene therapy and genome editing applications^2,6^, recent advances have been reported in the development of chemically defined cytokine-free human HSC expansion conditions that enable culture of the cells over a few weeks^12^. Remarkably, while these cultures enable more human HSCs to be preserved, we found that by adding 10 µM Lip-1, we could expand LT-HSCs by ~50-fold, while also expanding ST-HSCs and primitive progenitors (CD34^+^CD45RA^−^CD90^+^) after 3 weeks in these culture conditions (Fig. 2a–e and Extended Data Figs. 1b and 3a–f). Similar results were obtained when using the structurally distinct radical-trapping antioxidant Fer-1, further supporting the robustness and generalizability of our findings (Extended Data Fig. 3g–l). Our results reveal how even in conditions optimized for maximal HSC expansion^12^, the preservation of HSCs is probably suboptimal and can be further enhanced by inhibiting ferroptosis.

**Extended Data Fig. 4:**
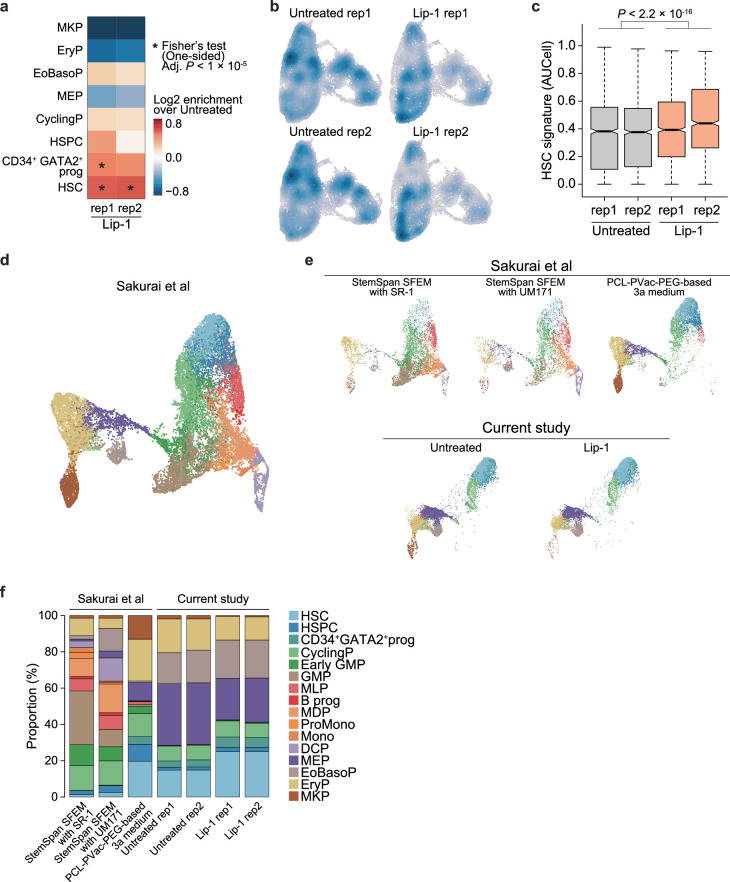
The current study aligns with previous analyses. **a**, Heatmap showing log2 enrichment of Lip-1-treated cells compared to the untreated control across cell types. Significance was calculated using a one-sided Fisher’s test and corrected using Benjamini & Hochberg (BH) method (*: adjusted P < 10^−5^) (n = 2 per condition). **b**, UMAP showing the cell density of control samples (left) and Lip-1 treated samples (right) (n = 2 per condition). **c**, Box plot showing the HSC signature score of Lip-1-treated HSCs compared to the untreated control, based on individual biological replicates (n = 2 per condition). The significance of differences in the two conditions was calculated based on a two-sided Wilcoxon rank-sum test. **d**, UMAP of scRNA-seq cells from Sakurai and collegues^12^ showing 10-day-expanded CD34^+^ cells in three culture conditions of PCL-PVAc-PEG-based 3a medium, StemSpan with SR-1 medium, or StemSpan with UM171 medium. **e**, UMAPs of the reference-based integration of scRNA-seq cells from Nakauchi’s group^12^ and the current study, illustrating the distribution of cells from each culture condition. **f**, Bar plots showing the proportion of scRNA-seq cells in individual culture conditions from Sakurai’s study^12^ and the current study.

While these results across a range of human HSC sources and culture approaches are promising, we have relied upon the fidelity of surface markers to quantify these cells. Such surface markers can display variability with some perturbations. Therefore, to further analyse the impact of inhibiting ferroptosis on human HSCs, we performed single-cell RNA sequencing (scRNA-seq) on CD34^+^CD45RA^−^CD90^+^ cells cultured with or without Lip-1 supplementation for 10 days in chemically defined conditions (Methods). We profiled a total of 29,096 cells collected from 2 different donors (with an average of 7,250 individual cells per condition) and annotated 10 cell clusters corresponding to known hematopoietic populations^30^, which were visualized using uniform manifold approximation and projection (UMAP) (Fig. 2f and Supplementary Table 1). The comparison of cluster composition and cellular density plots showed significant enrichment of molecularly defined HSCs in the Lip-1-treated condition (Fig. 2g–i and Extended Data Fig. 4a–c). Importantly, our findings of cell states were consistent with those observed in the original paper describing the use of chemically-defined culture conditions, demonstrating that the augmentation seen in our experiments arose in a setting consistent with the previous study^12^ (Extended Data Fig. 4d–f).

### Lip-1 augments in vivo repopulation capacity without signs of compromised haematopoiesis and does not cause detectable genotoxicity

**Fig. 3:**
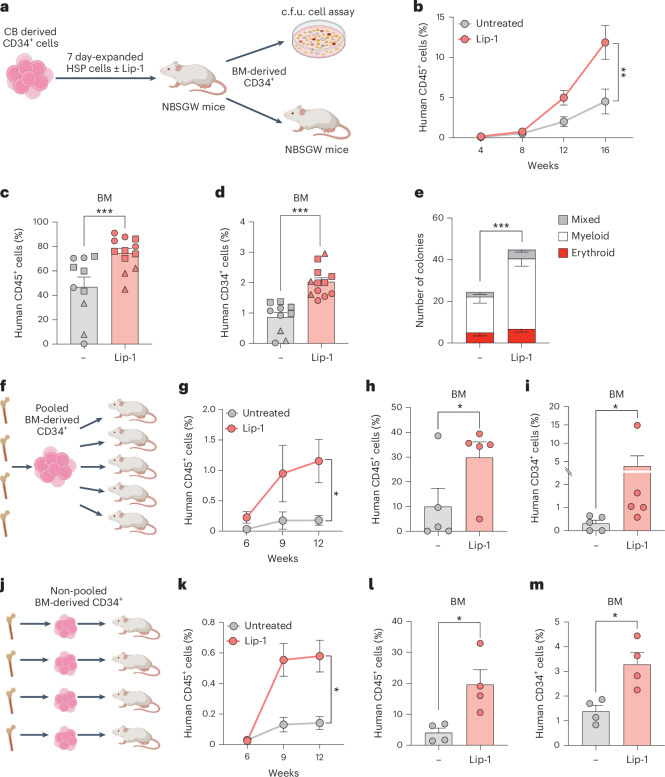
Lip-1 supplementation improves CB-HSPC in vivo repopulation capacity. **a**, CB-derived HSPCs from three independent donors (indicated by different symbol shapes: donor 1, circles; donor 2, squares; donor 3, triangles) were split into two groups and cultured for 7 days in a cytokine-free expansion medium either with or without Lip-1. Each condition was then transplanted into separate NBSGW recipient mice. At 16 weeks post-transplant, CD34⁺ cells were collected from the BM and either transplanted into secondary NBSGW recipients or seeded for c.f.u. cell assays. **b**,**c**, The percentage of human CD45^+^ cells measured at the indicated time points in the peripheral blood (**b**) and at 16 weeks in the BM (**c**) of mice transplanted with HSPCs cultured as indicated (donor 1: *n* mice = 4, 4; donor 2: *n* mice = 4, 5; donor 3: *n* mice = 2, 3). Linear mixed-effects model test (calculated at the last time point for PB) (*P* = 0.00136 (**b**) and 0.000812 (**c**)). **d**, The percentage of human CD34^+^ cells measured at 16 weeks in the BM of mice transplanted with HSPCs cultured as indicated (donor 1: *n* mice = 4, 4; donor 2: *n* mice = 4, 5; donor 3: *n* mice = 2, 3). Linear mixed-effects model test (*P* = 0.000008). **e**, The number of colonies formed by BM-derived CD34^+^ cells purified from mice in **c** at 16 weeks post-transplantation. Data are shown for two independent donors (donor 1: *n* mice = 4, 4; donor 2: *n* mice = 4, 5). Linear mixed-effects model test was computed on total colony numbers (*P* *=* 0.000407). **f**, A schematic illustrating that CD34⁺ cells collected from the BM of multiple primary mice within the same experimental group were pooled, and equal numbers of cells were transplanted into individual secondary recipients. **g**, The percentage of human CD45⁺ cells in the peripheral blood of secondary recipients transplanted with HSPCs collected from the BM of primary donors shown as circles in **c**, monitored up to 12 weeks (*n* mice = 5 per group). Mann–Whitney test (*P* = 0.0159). **h**, The percentage of human CD45^+^ cells measured at 12 weeks in the BM of mice in **g** (*n* mice = 5 per group). Mann–Whitney test (*P* = 0.037). **i**, The percentage of human CD34^+^ cells measured at 12 weeks in the BM of mice in **g** (*n* mice = 5 per group). Mann–Whitney test (*P* = 0.0238). **j**, A schematic illustrating that CD34⁺ cells collected from the BM of each individual primary mouse were transplanted into a single secondary recipient, following a one-donor–one-recipient approach. **k**, the percentage of human CD45⁺ cells in the peripheral blood of secondary recipients transplanted with HSPCs collected from the BM of primary donors shown as squares in **c**, monitored up to 12 weeks (*n* mice = 5 per group). Mann–Whitney test (*P* = 0.0286). **l**, The percentage of human CD45^+^ cells measured at 12 weeks in the BM of mice in **k** (*n* mice = 4 per group). Mann–Whitney test (*P* = 0.0286). **m**, The percentage of human CD34^+^ cells measured at 12 weeks in the BM of mice in **k** (*n* mice = 4 per group). Mann–Whitney test (*P* = 0.0286). Each dot represents an independent mouse, and each shape corresponds to a different donor. All the data are presented as mean ± s.e.m. Unless otherwise specified, a two-sided statistical test was used. *P < 0.05, **P < 0.01, ***P < 0.001 and ****P < 0.0001. Panels **a**, **f** and **j** created with BioRender.com. Source data

**Extended Data Fig. 5:**
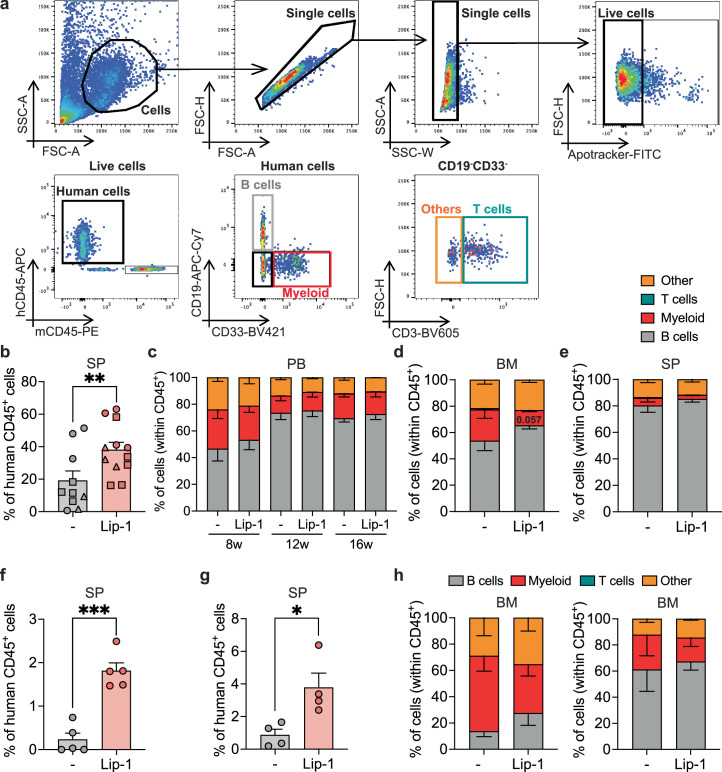
Lip-1 supplementation does not impact hematopoietic lineages in several organs. **a**, Representative plots and gating strategy for *in vivo* studies. Firstly, we gated on live cells (Apotracker^−^), then human CD45^+^ cells were distinguished from murine cells (mCD45^+^). Within this gate, we further gated on CD19^+^ (B cells) and CD33^+^ (Myeloid cells), subsequently, in the double negative gate, we identified CD3^+^ (T cells). Cells negative for all the lineage markers used were defined as “others”. **b**, Percentage of human CD45^+^ cells measured at 15w in the SP of mice in Fig. 3b transplanted with HSPCs cultured as indicated (Donor 1: n of mice=4,4; Donor 2: n of mice=4,5; Donor 3: n of mice=2,3). Linear mixed-effects model test (p-value: 0.00353). **c-e**, Percentage of B cells, Myeloid, T cells, and other cells within the (**c**) PB, (**d**) BM, and (**e**) SP of mice transplanted with HSPCs cultured as indicated (Donor 1: n of mice=4,4; Donor 2: n of mice=4,5; Donor 3: n of mice=2,3). Linear mixed-effects model test (p-value: (d) 0.0579). **f**, Percentage of human CD45^+^ cells measured at 12 weeks in the SP of mice in Fig. 3g (n of mice=5 per group). Mann-Whitney test (p-value: 0.000152). **g**, Percentage of human CD45^+^ cells measured at 12 weeks in the SP of mice in Fig. 3k (n of mice=4 per group). Mann-Whitney test (p-value: 0.0286). **h**, Percentage of B cells (CD19^+^), Myeloid (CD33^+^), T cells (CD3^+^), and other cells within the BM of mice in Fig. 3g (left panel) and Fig. 3k (right panel) (left: n of mice=5 per group; right: n of mice=4 per group). Each dot represents an independent mouse, and each shape corresponds to a different donor. All the data are presented as Mean ± SEM. Unless otherwise specified, a two-sided statistical test was used. *p < 0.05; **p < 0.01; ***p < 0.001; ****p < 0.0001. Source data

While our earlier results suggest promise for inhibiting ferroptosis as a strategy to preserve and expand more human HSCs ex vivo across different culture approaches, we sought to ensure that this would preserve appropriate stem cell functionality and not result in aberrant haematopoiesis. Following 7 days of culture with or without Lip-1 in chemically-defined conditions, CB-derived haematopoietic stem and progenitor cells (HSPCs) from three different donors were transplanted into the NOD.Cg-*Kit*^*W*-*41J*^ *Tyr*^+^ *Prkdc*^*scid*^ *Il2rg*^*tm1Wjl*^/ThomJ (NBSGW) strain of immunodeficient and *Kit*-mutant mouse recipients^16,22,31^ (Fig. 3a). Once long-term engraftment was achieved at 16 weeks post-transplantation from equivalent starting cell numbers, we observed greater repopulation capacity over time in the peripheral blood as well as in the BM and spleen with the Lip-1-treated cells (Fig. 3b,c and Extended Data Fig. 5a,b). Analysis of haematopoiesis in these engrafted mice showed a similar composition of myeloid and lymphoid cells without any notable alterations among diverse haematopoietic lineages upon Lip-1 treatment (Extended Data Fig. 5c–e). Importantly, the mice did not display any signs of impaired haematopoiesis or malignancy.

The NBSGW recipients transplanted with the Lip-1-treated HSPCs showed more CD34^+^ HSPCs in the BM after long-term reconstitution (Fig. 3d). Moreover, when we enriched for BM-derived CD34^+^ cells at 16 weeks post-transplant and tested their clonogenic potential, we observed a higher number of colonies generated by the Lip-1 groups, with colonies representative of all major lineages that were similar to controls across all donors examined (Fig. 3e).

We also tested the serial repopulating capacity of long-term HSCs by performing secondary transplants with BM-derived CD34^+^ cells from the primary CB-transplanted mice (Fig. 3a). We initially pooled CD34⁺ HSPCs collected from the BM of multiple primary recipients and transplanted equal numbers of cells into secondary mice (Fig. 3f). To ensure that the results were not confounded by specific engrafted samples, we also conducted secondary xenografts using transplantation of one donor to one recipient in a non-pooled manner, which yielded similar results (Fig. 3j). Importantly, despite the different approaches, we observed higher human haematopoietic engraftment among the Lip-1-treated group across all organs analysed, rigorously demonstrating the more robust HSC expansion by mitigating ferroptosis (Fig. 3g,h,k,l and Extended Data Fig. 5f,g). Moreover, while lineage output was similar between groups, the Lip-1-treated cohort displayed a higher fraction of stem and progenitor cells (Fig. 3i,m and Extended Data Fig. 5h).

**Fig. 4:**
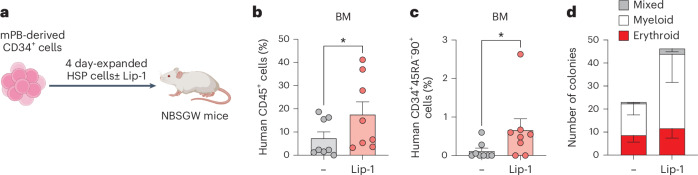
Improved engraftment and progenitor output of adult-derived HSPCs upon Lip-1 treatment. **a**, mPB-derived HSPCs from a single donor were split into two groups and cultured for 4 days in a serum-free expansion medium either with or without Lip-1. Each condition was then transplanted into eight separate NBSGW recipient mice. **b**, The percentage of human CD45^+^ cells measured at 12 weeks in the BM of mice transplanted with HSPCs cultured as indicated (*n* mice = 8 per group). Mann–Whitney test (*P* *=* 0.0415). **c**, The percentage of human CD34^+^45RA^−^90^+^cells measured at 12 weeks in the BM of mice transplanted with HSPCs cultured as indicated (*n* mice = 8 per group). Mann–Whitney test (*P* = 0.0284). **d**, The number of colonies formed by BM-derived CD34^+^ cells purified from mice in **b** at 12 weeks post-transplantation (*n* mice = 8 per group). Each dot represents an independent mouse and all the data are represented as mean ± s.e.m. Unless otherwise specified, a two-sided statistical test was used. *P < 0.05, **P < 0.01, ***P < 0.001 and ****P < 0.0001. Panel **a** created with BioRender.com. Source data

To extend these results to other cell sources and culture methods, we cultured mPB-derived CD34⁺ HSPCs for only 4 days in serum-free culture conditions, akin to commonly applied approaches in gene and cell therapy protocols. Consistent with the findings from transplanting CB-derived HSPCs in chemically defined conditions, similar results were obtained by transplanting shorter-term expanded mPB-derived cells into NBSGW mice (Fig. 4a). At 12 weeks post-transplantation, we observed higher human chimerism in the Lip-1-treated group, with a notable increase in the frequency of more primitive progenitor cells (Fig. 4b,c). BM-derived CD34⁺ cells obtained from the Lip-1 group exhibited greater colony output in methylcellulose assays (Fig. 4d). Collectively, these results demonstrate that Lip-1 treatment consistently improves the long-term repopulating capacity of both CB and adult-derived HSPCs, even in short-term serum-free cultures, validating its potential for clinically relevant HSC expansion.

**Extended Data Fig. 6:**
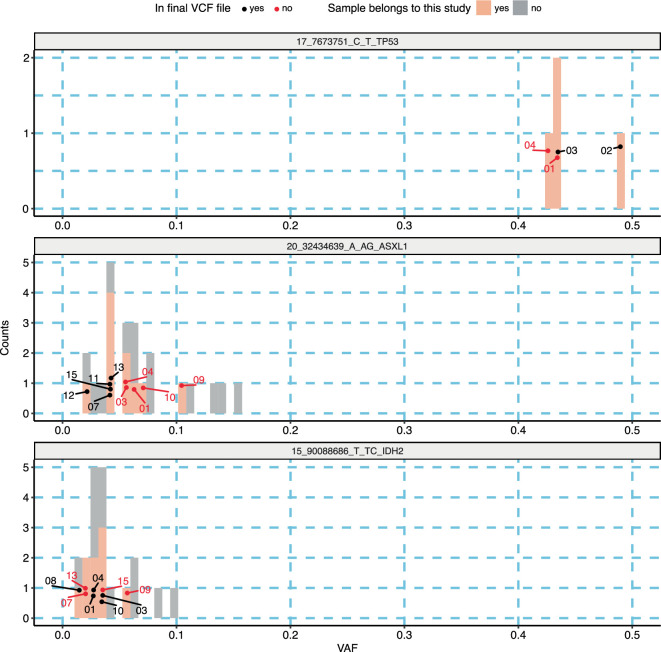
Targeted sequencing does not report any reliable CHIP-associated mutations. From VANTAGE analysis, three variants were reported as recurrent. From the top: a missense mutation in *TP53* with a VAF close to 0.5 is a germline variant, being present in all four samples from donor CB-52. *ASXL1* and *IDH2* genes were found to have single-base insertions in homopolymeric tracts; as they are present across multiple independent samples from this study and healthy donor bone marrow samples from an unrelated study (gray bars), they were considered as PCR artifacts.

**Extended Data Fig. 7:**
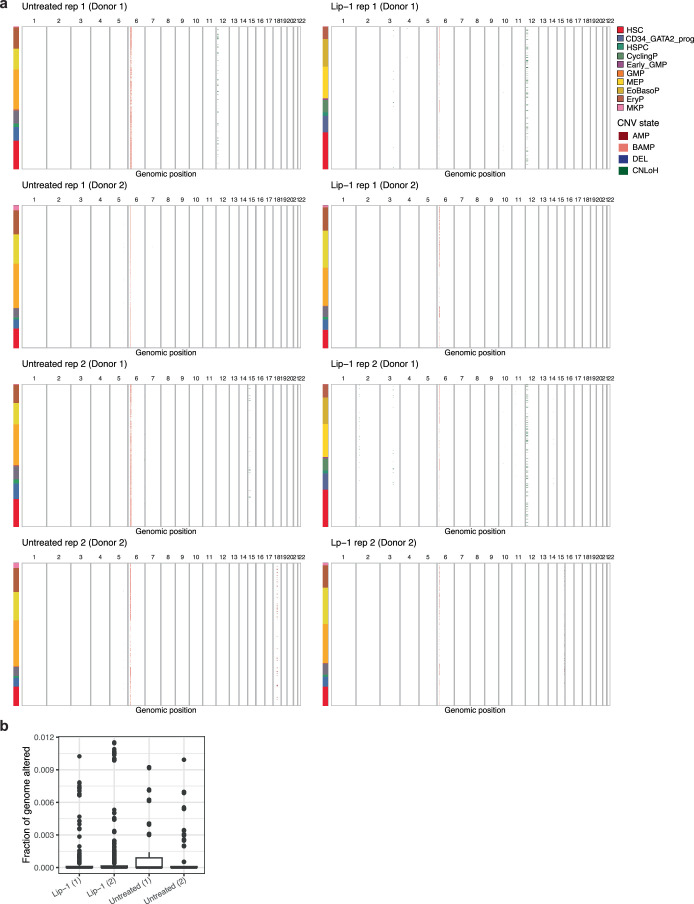
Analysis of copy number variation in scRNA-seq data. **a**, Copy number profiles of HSPCs cultured in presence or absence of Lip-1 inferred from scRNA-seq using Numbat^33^. Each row represents one cell and each line (colored by CNV type) represents a distinct inferred CNV in the cell. Events were filtered by posterior CNV probability > 0.95. **b**, Fraction of genome altered by expression-inferred CNV in each sample. Only autosomes are included in the analysis. Events were filtered by posterior CNV probability > 0.95. Replicates from each donor were combined for this analysis.

While our results suggest that haematopoiesis is preserved and appears normal, even after transplantation, we sought to ensure that there were no signs of aberrant haematopoiesis. To assess genotoxicity in depth, we profiled 137 amplicons covering >95% of clonal haematopoiesis of indeterminate potential mutations^32^ across three different donors in samples cultured ex vivo with or without Lip-1 over several weeks (the same samples were analysed after 1 and 3 weeks of culture). While a single germline benign polymorphism was detected in *TP53* in one donor (Methods), no somatic variants were identified and no signs of clonal expansions were noted in this analysis (Extended Data Fig. 6 and Supplementary Table 2). We also examined the scRNA-seq data we obtained to assess for major structural variants or aneuploidies using a sensitive tool for haplotype-aware copy number analyses, Numbat^33^. We observed no signs of detectable copy number variation in Lip-1-treated haematopoietic cells in comparison with controls (Extended Data Fig. 7a,b). Collectively, these results strongly support the contention that while blockade of ferroptosis with Lip-1 improves human HSC expansion ex vivo, this does not appear to promote genotoxicity in the cells.

### Dissecting mechanisms of Lip-1 treatment impact upon human HSCs

**Fig. 5:**
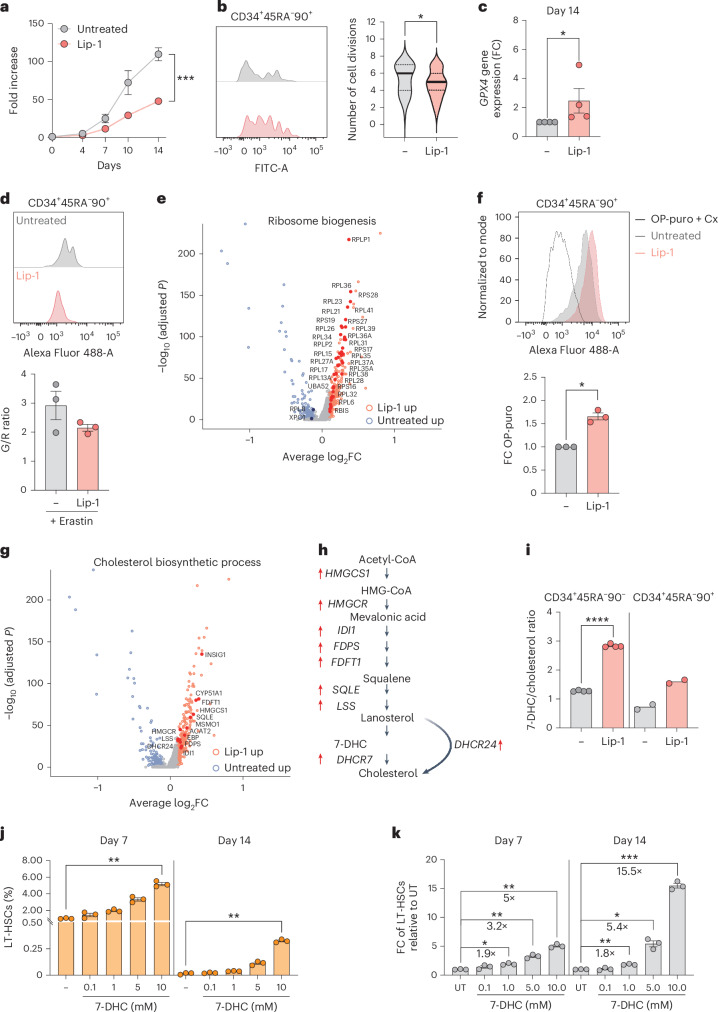
Lip-1 reduces HSPC proliferation and lipid peroxidation while increasing GPX4 expression as well as ribosomal and cholesterol synthesis pathways. **a**, A growth curve of mPB-derived HSPCs cultured for up to 14 days in a serum-free expansion medium in the presence or absence of Lip-1. The fold increase was calculated as the number of cells counted at an indicated time point over the number of cells at day 0 (*n* = 5 per condition). Student’s *t*-test (*P* = 0.000145). **b**, The number of cellular divisions performed by mPB-derived CD34^+^CD45RA^−^CD90^+^ on day 14. Up to 180 cells were analysed. Mann–Whitney test (*P* = 0.0123). **c**, The relative expression of the *GPX4* gene at day 14. The fold change was calculated relative to the untreated condition (*n* = 4 per condition). Student’s *t*-test (*P* = 0.0465). **d**, A representative flow cytometric histogram (top) of oxidized BODIPY dye of CD34^+^CD45RA^−^CD90^+^ treated with 25 µM Erastin for 6 h on day 14. The quantification of lipid peroxidation level (bottom) is measured as the ratio of oxidized (green (G) signal) and non-oxidized (red (R) signal) BODIPY dye (G/R ratio) (*n* = 3 per condition). **e**, A volcano plot showing the differential expression between untreated and Lip-1 cells, highlighting genes involved in ribosome biogenesis. Wilcoxon rank-sum test, *P* values were adjusted using the Benjamini–Hochberg method. **f**, A representative flow cytometric histogram (top) showing OP-puro incorporation to assess protein synthesis in mPB-derived CD34⁺CD45RA⁻CD90⁺ cells cultured for 4 days in serum-free expansion medium with or without Lip-1. The quantification (bottom) of fold change in translation rates relative to control is shown. Cycloheximide (Cx) was used as a negative control to block protein synthesis (*n* = 3 per condition). One sample *t*-test (*P* = 0.0129). **g**, A volcano plot showing the differential expression between untreated and Lip-1 cells, highlighting genes involved in the cholesterol biosynthetic process. Wilcoxon rank-sum test, *P* values were adjusted using the Benjamini–Hochberg method. **h**, A schematic representation of the cholesterol production pathway. The enzymes responsible for the specific conversion are in italics and the arrows indicate the trend of their expression in the Lip-1 condition. **i**, The relative quantification of 7-DHC and cholesterol concentrations in CD34^+^CD45RA^−^CD90^−^ and CD34^+^CD45RA^−^CD90^+^ cells sorted from untreated or Lip-1 samples (*n* = 4, 4, 2, 2). Student’s *t*-test (*P* = 3.81 × 10^−9^). **j**,**k**, The percentage (**j**) and fold change (**k**) of LT-HSCs (CD34^+^CD45RA^−^CD133^+^CD90^+^EPCR^+^ITGA^+^) in mPB cells at day 7 and 14 cultured in serum-free medium supplemented with the indicated doses of 7-DHC (*n* = 3 per condition). Friedman test followed by Dunn’s multiple comparison test (**j**) (*P* = 0.0078, 0.0078) and one sample *t*-test (**k**) (*P* = 0.0108, 0.0071, 0.0022, 0.0058, 0.0132, 0.0008). Each dot represents an independent donor unless otherwise indicated and all the data are presented as mean ± s.e.m. Unless otherwise specified, a two-sided statistical test was used. *P < 0.05, **P < 0.01, ***P < 0.001 and ****P < 0.0001. Source data

**Extended Data Fig. 8:**
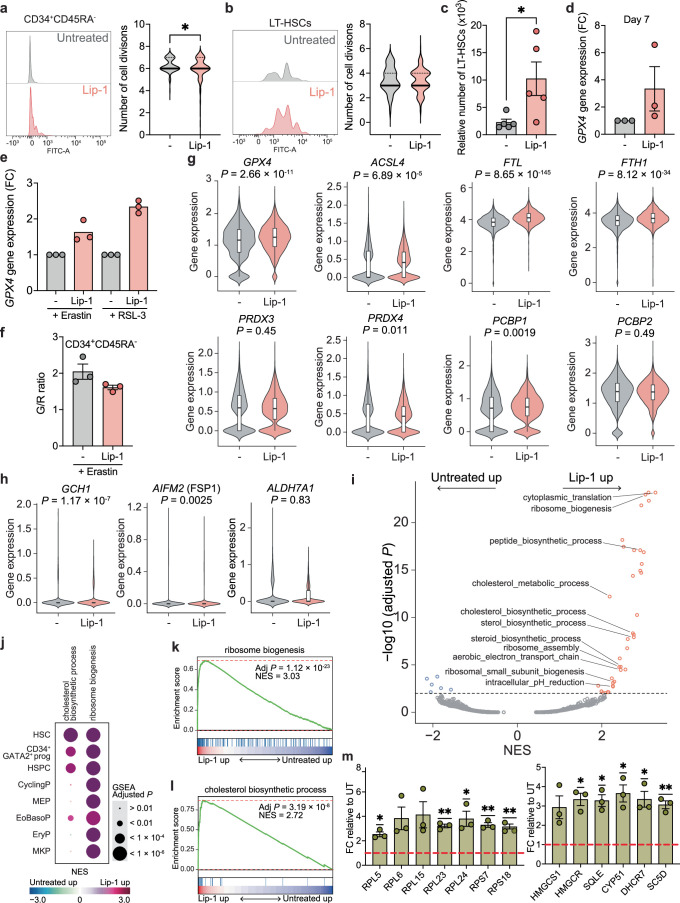
HSPCs cultured in the presence of Lip-1 exhibit slower cell proliferation and higher expression of ferroptosis protective, ribosome-, and cholesterol-related genes. **a**, Number of cellular divisions performed by mPB-derived CD34^+^CD45RA^−^ on day 14. More than 4×10^4^ cells were analysed from 2 independent donors. Mann-Whitney test (p-value: 0.0204). **b**, Number of cellular divisions performed by mPB-derived LT-HSCs (CD34^+^CD45RA^−^CD133^+^CD90^+^EPCR^+^ITGA3^+^) on day 14. Up to 120 cells were analyzed from 2 independent donor. **c**, Number of mPB-derived LT-HSCs calculated at day 14. Cell counts were estimated based on an initial population of 10^5^ cells and multiplying the fold increase in Fig. 5a by the percentage of LT-HSCs at the corresponding time point (n = 5 per condition). Mann-Whitney test (p-value: 0.0159). **d**, Relative expression of *GPX4* gene at day 7. Fold change was calculated relatively to untreated condition (n = 3 per condition). **e**, Relative expression of *GPX4* gene in HSPCs cultured with or without Lip-1 and treated with 25 µM Erastin or RSL-3 for 6 h at day 14. Fold change was calculated relative to untreated condition (n = 3 technical replicates). **f**, Quantification of lipid peroxidation level in CD34^+^CD45RA^−^ treated with 25 µM Erastin for 6 h on day 14 measured by the ratio of oxidized and non-oxidized BODIPY dye (n = 3 per condition). **g**, Representative violin plots of iron transport, storage, and metabolism gene expression in untreated or Lip-1 transcriptionally defined HSCs (n = 2 per condition). **h**, Representative violin plots of *AIFM2*, *GCH1*, and *ALDH7A1* gene expression in untreated or Lip-1 transcriptionally defined HSCs (n = 2 per condition). **i**, Scatter plot showing the GSEA-enriched biological pathways based on RNA expression between Lip-1 and untreated group using the 6,035 gene sets from GO Biological Process 2021 database (n = 2 per condition). **j**, GSEA bubble plot showing differential pathways (cholesterol biosynthetic process and ribosome biogenesis) between Lip-1-treated and untreated cells across cell types (n = 2 per condition). **k, l**, GSEA enrichment plots showing that the genes involved in (**k**) ribosome biogenesis and (**l**) cholesterol biosynthetic process are significantly upregulated in the Lip-1 group (n = 2 per condition). The significance was calculated based on an adaptive multi-level split Monte-Carlo scheme. **m**, Relative expression of ribosomal protein genes (left) and cholesterol biosynthesis-related genes (right) in CD34^+^CD45RA^−^CD90^+^ sorted HSPCs cultured in cytokine-free expansion medium with or without Fer-1 for 10 days. Fold change was calculated relatively to untreated condition (n = 3 per condition). One sample t-test (p-values: (left) 0.0161; 0.0921; 0.0997; 0.0044; 0.0458; 0.0069; 0.0085; (right) 0.082; 0.0178; 0.0172; 0.0268; 0.0287; 0.0097). Each dot represents an independent donor unless otherwise indicated and all the data are presented as Mean ± SEM. Unless otherwise specified, a two-sided statistical test was used. *p < 0.05; **p < 0.01; ***p < 0.001; ****p < 0.0001. Source data

While our findings build upon our prior studies in the context of BM failure^16^, we wanted to more fully understand the mechanisms underlying the improved expansion of HSCs observed by preventing ferroptosis. Interestingly, when cells were cultured with Lip-1 (at 10 µM), we observed overall decreased cell expansion in the cultures (Fig. 5a and Extended Data Fig. 8a) and, concomitantly, we found that HSC-enriched fractions (CD34^+^CD45RA^−^CD90^+^) underwent fewer divisions (Fig. 5b), with a similar trend seen among phenotypic LT-HSCs (Extended Data Fig. 8b). While this could be attributable to either a direct slowing of cell divisions or improved maintenance of LT-HSCs, the numbers of LT-HSCs observed in the cultures were higher upon Lip-1 treatment (Extended Data Fig. 8c). Therefore, ferroptosis prevention helps to better preserve and expand bona fide LT-HSCs that would generally fail to be appropriately supported in such ex vivo cultures.

Consistent with prior studies, Lip-1 treatment was associated with increased expression of the key ferroptosis protective gene *GPX4* for up to 14 days in serum-free cultures (Fig. 5c and Extended Data Fig. 8d). Notably, even after the induction of ferroptosis by the molecules erastin and RSL-3 (refs. ^18,28^), Lip-1 supplementation protected human HSPCs with higher *GPX4* expression and subsequently decreased lipid peroxidation in progenitor and HSC-enriched subpopulations (Fig. 5d and Extended Data Fig. 8e,f). Assessment of the scRNA-seq data from molecularly defined HSC populations showed an upregulation of *GPX4* and iron-binding genes, all of which would be expected to protect against ferroptosis, as well as reductions in the expression of genes involved in oxidant detoxification, as would be expected under reduced oxidative stress (Extended Data Fig. 8g). Moreover, other major ferroptosis-regulating antioxidant genes^34–36^—*AIFM2* (also known as *FSP1*), *GCH1* and *ALDH7A1*—were expressed at low or undetectable levels in human HSCs (Extended Data Fig. 8h). These findings support the notion that ferroptosis inhibition in human HSCs may be primarily mediated through the GPX4 axis, while other ferroptosis regulators may have a less critical role in these cells under standard ex vivo culture conditions.

While Lip-1 and Fer-1 are well characterized to function as radical trapping antioxidants within the cell membrane that thereby prevent lipid peroxidation^17^, we hypothesized that there might be secondary adaptations in human HSCs that promote further protection from ferroptosis and enable improved HSC expansion. To examine this possibility, we queried the upregulated gene sets from the molecularly defined HSCs in the scRNA-seq data and unexpectedly found an enrichment of only two major pathways: ribosomal biogenesis/translation regulation and cholesterol metabolism (Extended Data Fig. 8i). Notably, the upregulation of cholesterol biosynthetic pathways was primarily confined to the HSC compartment, suggesting a cell type-specific transcriptional response in the setting of Lip-1 treatment (Extended Data Fig. 8j).

In particular, a substantial number of ribosomal protein genes—including both large and small subunit components—were significantly upregulated, suggesting a coordinated increase in ribosome production (Fig. 5e and Extended Data Fig. 8k). To directly assess whether protein synthesis rates were altered, we labelled CD34⁺CD45RA⁻CD90⁺ cells with *O*-propargyl-puromycin (OP-puro), a puromycin analogue that gets incorporated into and terminates nascent polypeptide chains, to assess protein synthesis rates^16,37,38^. Lip-1-treated HSCs had a ~1.5-fold increase in protein synthesis in comparison with untreated cells (Fig. 5f). While HSCs are known to typically maintain low and highly regulated protein synthesis rates^37,38^, our prior work has shown that slightly increased translation within human HSCs can protect from ferroptosis^16^, aligning with our current observations.

The upregulation of cholesterol biosynthesis was unanticipated and noteworthy given recent studies demonstrating a role for intermediates in this pathway, particularly B-ring-unsaturated sterols such as 7-dehydrocholesterol (7-DHC), in protecting cells from ferroptosis^39,40^. Remarkably, we noted upregulated gene expression for almost all components of the cholesterol biosynthesis pathway^41^ (Fig. 5g,h and Extended Data Fig. 8l). Importantly, upregulation of ribosomal protein and cholesterol biosynthesis genes were also observed upon Fer-1 treatment, suggesting that these gene expression changes reflect a general adaptation of HSCs that are protected from ferroptosis (Extended Data Fig. 8m).

**Extended Data Fig. 9:**
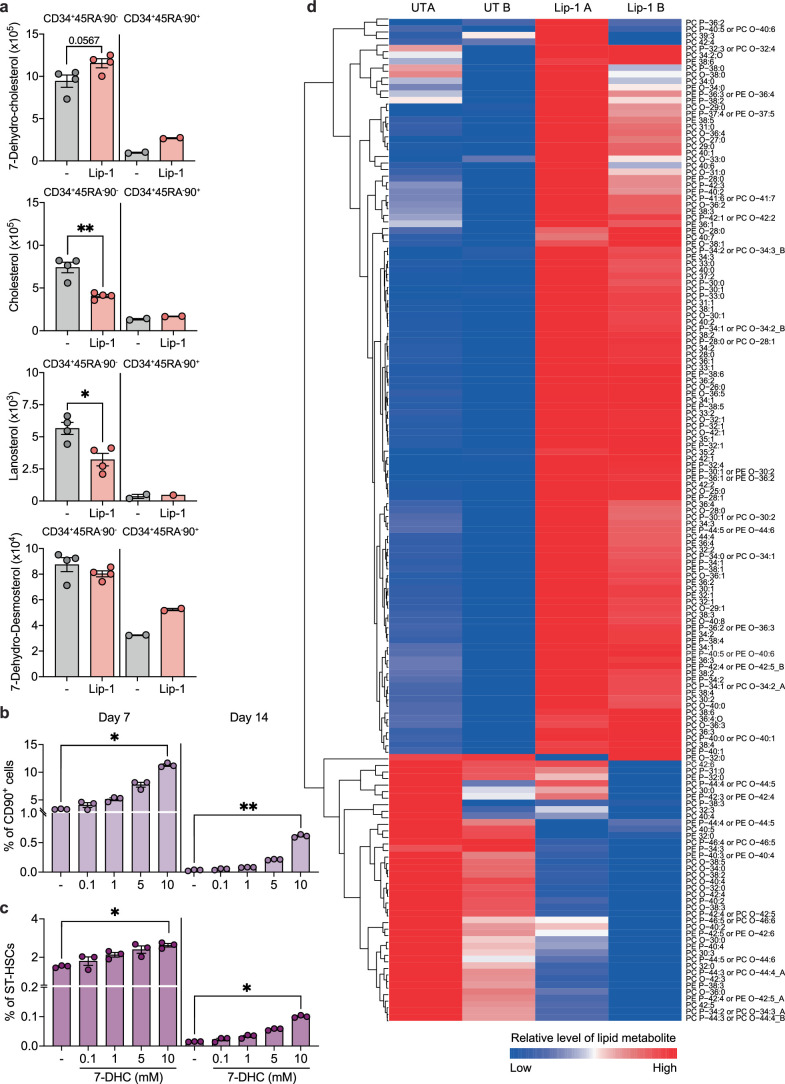
Trends of cholesterol, other intermediates, and phospholipids in HSCs cultured in the presence of Lip-1. **a**, Concentration of (from the top) 7-DHC, cholesterol, lanosterol, and 7-dehydro-desmosterol in sorted CD34^+^CD45RA^−^CD90^−^ and CD34^+^CD45RA^−^CD90^+^ cells cultured with or without Lip-1 (n = 4,4,2,2). Student’s t-test (p-values: 0.0567; 0.0115; 0.00184). **b, c**, Percentage of (**b**) primitive HSPCs (CD34^+^CD45RA^−^CD90^+^) and (**c**) ST-HSCs (CD34^+^CD45RA^−^CD133^+^CD90^+^EPCR^+^) in mPB cells at day 7 and 14 cultured in serum-free medium supplemented with the indicated doses of 7-DHC (n = 3 per condition). Friedman test (p-values: (**c**) 0.018; 0.0078; (**d**) 0.018; 0.018). **d**, Heatmap describing the relative levels of phospholipids in CD34^+^CD45RA^−^CD90^+^ cells cultured with or without Lip-1, showing a reduction of phospholipids with polyunsaturated fatty acids and polyunsaturated plasmalogens. Each dot represents an independent donor unless otherwise indicated and all the data are presented as Mean ± SEM. Unless otherwise specified, a two-sided statistical test was used. *p < 0.05; **p < 0.01; ***p < 0.001; ****p < 0.0001. Source data

To examine the impact of these alterations, we performed lipidomic analysis on sorted HSC enriched (CD34^+^CD45RA^−^CD90^+^) and other haematopoietic progenitors (CD34^+^CD45RA^−^CD90^−^) in chemically defined culture conditions. While the levels of cholesterol and other measured intermediates were only slightly elevated or unchanged in the HSC-enriched population and were reduced in progenitors, the amount of 7-DHC was substantially increased, consistent with a state that can robustly protect cells from lipid peroxidation and resultant ferroptosis (Fig. 5i and Extended Data Fig. 9a). Notably, some of the observed upregulation in 7-DHC levels could be attributed to increased radical scavenging by Lip-1 and Fer-1, which thereby preserve greater 7-DHC levels in the membrane, although the observed gene expression changes suggest at least some impact from altered cholesterol biosynthesis. Direct supplementation with 7-DHC expanded HSC-enriched subpopulations, with an increase in primitive progenitors, as well as ST- and LT-HSCs, resembling the effect of Lip-1 or Fer-1 addition (Fig. 5j,k and Extended Data Fig. 9b,c). Furthermore, we also examined the diversity of phospholipids within human HSCs cultured with Lip-1 and observed a reduction of phospholipids with polyunsaturated fatty acids and plasmalogens, which would reduce the propensity for a cell to undergo ferroptosis^42^ (Extended Data Fig. 9d). These results point to a remarkable metabolic adaptation within cultured human HSCs that increases expression of the protein synthesis machinery and alters cholesterol biosynthesis, while also reducing polyunsaturated phospholipids, to protect from ferroptosis.

### Prevention of ferroptosis to improve HSC yields with genome engineering

Although our earlier results support the notion that human HSC expansion with different cell sources and culture approaches can be improved by preventing ferroptosis, one of the most immediate applications of this advance would be in the emerging clinical use of genome engineering approaches in HSCs cultured ex vivo. This is particularly important as genome editing of human HSCs involves additional manipulations, such as the introduction of recombinant Cas9 protein or derivatives, as well as the electroporation of cells, which might promote further cell loss.

**Fig. 6:**
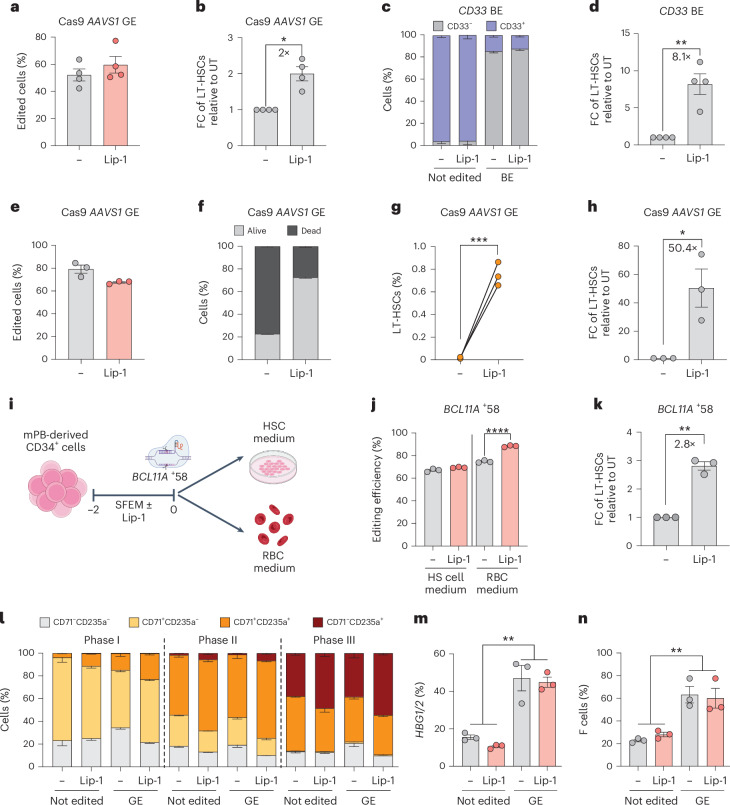
Lip-1 enhances the expansion of genome-engineered HSCs without impacting editing or phenotypic outcomes. **a**, The percentage of gene-edited (GE) HSPCs on day 14 (*n* = 4 per condition). **b**, The fold change of LT-HSCs at day 14 was calculated as the ratio of Lip-1 group % LT-HSCs/untreated group %L T-HSCs (*n* = 4 per condition). One sample *t*-test (*P* = 0.0146). **c**, The percentage of base-edited (BE) cells expressing the CD33 surface marker (*n* = 3 per condition). **d**, The fold change of LT-HSCs at day 14 was calculated as the ratio of Lip-1 group % LT-HSCs/untreated group %L T-HSCs (*n* = 4 per condition). One sample *t*-test (*P* = 0.0139). **e**, The percentage of edited HSPCs on day 21 (*n* = 3 per condition). **f**, The cell viability analysis of CB-HSPCs on day 14 was conducted using flow cytometry. The Apotracker probe allowed the detection of dead cells (*n* = 3 per condition). **g**, The percentage of LT-HSCs (CD34^+^CD45RA^−^CD133^+^CD90^+^EPCR^+^ITGA3^+^) at day 21 (*n* = 3 per condition). Student’s *t*-test (*P* = 0.000256). **h**, The fold change of LT-HSCs at day 21 was calculated as the ratio of Lip-1 group % LT-HSCs/ untreated group %L T-HSCs (*n* = 3 per condition). One-sample *t*-test (*P* = 0.0211). **i**, The experimental workflow of in vitro erythroid differentiation experiments. mPB-derived HSPCs were cultured for 2 days in a serum-free expansion medium in the presence or absence of Lip-1. Cells were Cas9-edited targeting *BCL11A* enhancer (DHS +58) and immediately seeded either in red blood cell (RBC) differentiation medium or HSC medium. **j**, The percentage of edited cells on day 7 of HSC culture or day 12 of RBC differentiation (*n* = 3 per condition). Student’s *t*-test (*P* = 0.000053). **k**, The fold change of LT-HSCs at day 21 was calculated as the ratio of Lip-1 group % LT-HSCs/untreated group % LT-HSCs (*n* = 3 per condition). One-sample *t*-test (*P* = 0.0067). **l**, Analysis of culture composition during different phases of RBC differentiation (*n* = 3 per condition). **m**, The percentage of *γ*-globin genes (*HBG1*/*2*) at day 17 (phase III) of erythroid differentiation (*n* = 3 per condition). Kruskal–Wallis test (*P* = 0.0014). **n**, Fetal haemoglobin (HbF) levels measured in fetal red blood cells (F cells) by flow cytometry on day 17 (phase III) of erythroid differentiation (*n* = 3 per condition). Kruskal–Wallis test (*P* = 0.0028). Each dot represents an independent donor unless otherwise indicated and all the data are presented as mean ± s.e.m. Unless otherwise specified, a two-sided statistical test was used. *P < 0.05, **P < 0.01, ***P < 0.001 and ****P < 0.0001. Panel **i** created with BioRender.com. Source data

**Extended Data Fig. 10:**
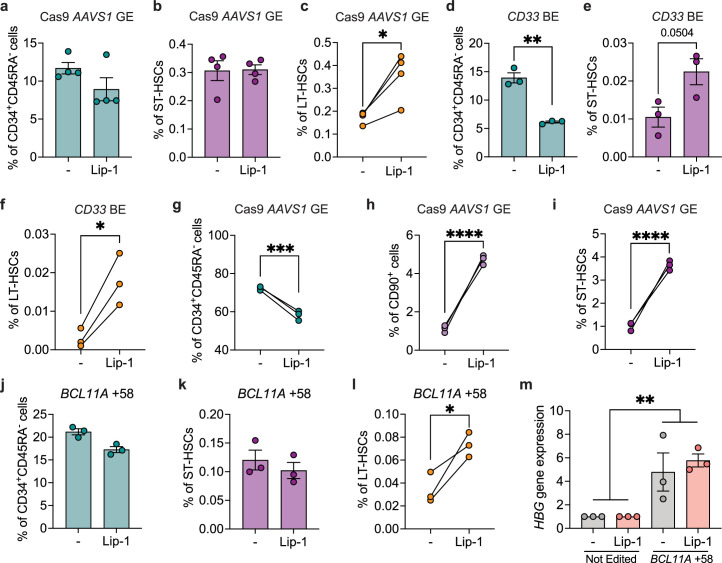
Culture composition of gene-edited HSPCs cultured with or without Lip-1 supplementation. **a-c**, Percentage of (**a**) CD34^+^CD45RA^−^, (**b**) ST-HSCs (CD34^+^CD45RA^−^CD133^+^CD90^+^EPCR^+^), and (**c**) LT-HSCs (CD34^+^CD45RA^−^CD133^+^CD90^+^EPCR^+^ITGA3^+^) in mPB-derived HSPCs edited by Cas9 targeting the *AAVS1* locus at day 14 (n = 4 per condition). Student’s t-test (p-value: 0.0158). **d-f**, Percentage of (**d**) CD34^+^CD45RA^−^, (**e**) ST-HSCs (CD34^+^CD45RA^−^CD133^+^CD90^+^EPCR^+^), and (**f**) LT-HSCs (CD34^+^CD45RA^−^CD133^+^CD90^+^EPCR^+^ITGA3^+^) in mPB-derived HSPCs edited by ABE8e targeting the *CD33* locus at day 14 (n = 3 per condition). Student’s t-test (p-values: (**d**) 0.001; (**e**) 0.0504; (**f**) 0.022). **g-i**, Percentage of (**g**) CD34^+^CD45RA^−^, (**h**) primitive HSPCs (CD34^+^CD45RA^−^CD90^+^), and (**i**) ST-HSCs (CD34^+^CD45RA^−^CD133^+^CD90^+^EPCR^+^) in CB-derived HSPCs cultured in cytokine-free medium and edited by Cas9 targeting the *AAVS1* locus at day 14 (n = 3 per condition). Student’s t-test (**p**-values: (**g**) 0.000826; (**h**) 0.0000365; (**i**) 0.0000787). **j-l**, Percentage of (**j**) CD34^+^CD45RA^−^, (**k**) ST-HSC (CD34^+^CD45RA^−^CD133^+^CD90^+^EPCR^+^), and (**l**) LT-HSC (CD34^+^CD45RA^−^CD133^+^CD90^+^EPCR^+^ITGA3^+^) in mPB-derived HSPCs edited by Cas9 targeting the *BCL11A* enhancer (DHS + 58) at day 14 (n = 3 per condition). Student’s t-test (p-value: 0.0169). **m**, Relative expression of *HBG* gene at day 17 (Phase III) of erythroid differentiation. Fold change was calculated relative to untreated condition (n = 3 per condition). Kruskal-Wallis test (p-value: 0.0045). Each dot represents an independent donor unless otherwise indicated and all the data are presented as Mean ± SEM. Unless otherwise specified, a two-sided statistical test was used. *p < 0.05; **p < 0.01; ***p < 0.001; ****p < 0.0001. Source data

First, we cultured mPB-derived HSPCs in serum-free cultures supplemented with Lip-1. Two days after thawing, HSPCs were nucleofected with Cas9 ribonucleoprotein complexes (RNP) pre-assembled with synthetic base-modified single-guide RNAs (sgRNAs) targeting the benign safe harbour adeno-associated virus integration site 1 (*AAVS1*) locus. After confirming that Lip-1 supplementation had no impact on editing efficiency, we observed a ~2-fold increase in the fraction of LT-HSCs, without notable changes in other subpopulations analysed (Fig. 6a,b and Extended Data Fig. 10a–c).

The CD33 surface protein is an attractive immunotherapy target for acute leukemia, but is also expressed on normal haematopoietic cells and therefore epitope editing of CD33 in HSCs would allow for the application of CD33-targeting immunotherapies concomitant with the continuation of normal haematopoiesis^43–46^. We therefore next examined what occurs when targeting the *CD33* locus with base editing. When using recombinant base editor protein-based RNPs, we observed a comparable loss of CD33 protein expression and level of editing, while there was an ~8-fold improved expansion of LT-HSCs with Lip-1 treatment (Fig. 6c,d and Extended Data Fig. 10d–f). These results demonstrate that the blockade of ferroptosis could improve HSC maintenance in the presence of various types of genome-engineering manipulations.

Next, we expanded our investigation to genome editing of CB-derived HSPCs cultured in chemically defined cytokine-free medium^12^. After a week in this culture, we introduced Cas9 RNPs and sgRNAs targeting the *AAVS1* locus again and obtained high and comparable levels of edited cells (Fig. 6e). Crucially, Lip-1 supplementation protected cells from loss post-editing and led to a marked expansion of HSC-enriched subpopulations (CD34^+^CD45RA^−^CD90^+^ cells), along with substantial increases in ST- and LT-HSCs, which exceeded 50-fold after 3 weeks of culture (Fig. 6f–h and Extended Data Fig. 10g–i).

Genome engineering of HSCs has now been clinically tested and approved for the treatment of sickle cell disease and β-thalassemia by using Cas9-based genome editing to disrupt the *BCL11A* +58 enhancer^6,47^. However, HSC numbers for these procedures are often limiting. We tested whether our approach of inhibiting ferroptosis could augment HSC retention in cultures akin to those applied clinically, while also showing similar editing efficiency. Cas9 RNPs targeting the *BCL11A* +58 enhancer were introduced into mPB-derived HSPCs, in a similar manner to what is being done clinically, and then HSCs were either retained in HSC expansion conditions or underwent erythroid differentiation^48^ (Fig. 6i). We observed efficient and comparable editing in the HSPC population, but surprisingly the edits were better preserved during erythroid differentiation of Lip-1-treated cells, suggesting that blocking ferroptosis could also better preserve edited cells (Fig. 6j). Importantly, LT-HSC expansion was improved by ~3-fold with minimal impact on other subpopulations (Fig. 6k and Extended Data Fig. 10j–l). In addition, early Lip-1 supplementation did not compromise erythroid differentiation capabilities of edited or unedited cells, while a similar extent of fetal haemoglobin induction was seen across all conditions (Fig. 6l–n and Extended Data Fig. 10m). These data from conditions that mimic what is being clinically applied in approved genome-editing therapies highlight a potential path for integration of ferroptosis blockade to improve HSC expansion in clinical settings.

## Discussion

While significant progress has been made to improve the maintenance and expansion of human HSCs ex vivo, this remains a significant challenge^7^. This is perhaps not all that surprising given that the minimal components present in culture fail to appropriately mimic the highly regulated niche within the BM in which these cells ordinarily reside throughout life^49–51^. Moreover, most ex vivo HSC culture protocols not only seek to maintain these cells that ordinarily undergo self-renewal once or twice a year in the BM, but also try to expand these cells over a short period in culture^4^. Therefore, it is likely that the existing culture methods fail to fully support human HSCs under the stressful conditions required for expansion. On the basis of our prior studies demonstrating a unique vulnerability of human HSCs to loss via ferroptosis^16^, here we explored whether mitigating ferroptosis in diverse and commonly applied culture conditions could improve the expansion of human HSCs. Remarkably, we found consistent impacts across a range of approaches without any signs of cell toxicity or indication that haematopoiesis is adversely impacted.

Our observations are likely to have an impact on clinical and translational efforts to maintain and expand HSCs ex vivo for gene therapy and genome-editing approaches in autologous settings, where collected cell numbers can be limiting, as well as in the setting of using allogeneic haematopoietic cell sources, where product availability can be limited for many individuals^52^. Given the ease with which radical-trapping antioxidants can be added to existing culture methods without compromising cell viability, this approach can be readily integrated into existing protocols. This will enable haematopoietic cell therapy approaches to be applied in more patients and require fewer cells to be collected, which could significantly advance treatment availability and options.

Although the focus of this work has been on advancing existing approaches for human HSC culture ex vivo, key biological insights have emerged, as well. Even though the regulation of cholesterol within HSCs has been studied^53,54^, we have uncovered how key intermediates of this pathway, such as 7-DHC, can enhance human HSC expansion in tandem with other metabolic alterations, such as reductions in polyunsaturated phospholipid levels. In addition, changes in cholesterol biogenesis enzymes may also impact other metabolic pathways, contributing to broader cellular adaptations. Studies building upon these observations could reveal critical new metabolic regulators involved in stem cell biology, paving the path towards additional advances in this field.

## Methods

### Primary cell culture

All experiments were conducted in accordance with relevant ethical regulations. The use of human HSPCs from CB and mPB was approved by the Institutional Review Board of Boston Children’s Hospital (protocol IRB-P00048735), and informed consent was obtained from all donors in accordance with the Declaration of Helsinki. Donors did not receive any form of financial or material compensation for their participation.

Human CD34^+^ HSPCs from mPB of healthy adults were obtained from the Cooperative Center of Excellence in Hematology at the Fred Hutchinson Cancer Research Center. After thawing, HSP cells were seeded at the concentration of 5 × 10^5^ cells ml^−1^ in serum-free StemSpan SFEM II medium (StemCell Technologies) supplemented with 1% l-glutamine (Thermo Fisher Scientific), 1% penicillin/streptomycin (Life Technologies), 1× CC100 (containing the cytokines FLT3L, SCF, IL-3 and IL-6; StemCell Technologies), 100 ng ml^−1^ recombinant thrombopoietin (PeproTech), and 35 nM UM171 (StemCell Technologies), as we have described previously^16,22,44^.

Human CD34^+^ HSPCs were also sourced from CB, obtained from the Dana-Farber Cancer Institute or Brigham and Women’s Hospital as discarded deidentified samples. CD34^+^ HSPCs were enriched from the CB by the EasySep Human Cord Blood CD34^+^ positive selection kit (StemCell Technologies) according to the manufacturer’s instructions. These cells were cultured either in serum-free StemSpan SFEM II medium (StemCell Technologies) with the aforementioned supplements or in Iscove’s modified Dulbecco’s medium (IMDM; Life Technologies). The IMDM was supplemented with 1% insulin–transferrin–selenium–ethanolamine (Life Technologies), 1% l-glutamine (Thermo Fisher Scientific), 1% penicillin/streptomycin (Life Technologies), 1 mg ml^−1^ polyvinyl alcohol (Sigma-Aldrich), 1 µM 740Y-P (MedChemExpress), 0.1 µM butyzamide (MedChemExpress) and 70 nM UM171. This latter culture method follows the protocol outlined in ref. ^12^. In this condition, human CB CD34^+^ cells were seeded at a density of 7 × 10^4^ to 1 × 10^5^ cells ml^−1^ in either 1 ml of medium per well in a 24-well plate or 5 ml of medium per well in a 6-well CellBind plate.

Where indicated, HSPCs were treated with Lip-1 (Caymen Chemicals), Fer-1 (MedChemExpress), sodium selenite (Sigma-Aldrich), β-ME (Thermo Fisher Scientific), RSL-3 (SelleckChem), Erastin (SelleckChem) and 7-DHC (Sigma-Aldrich). Unless otherwise specified, Lip-1 was used at 10 µM and Fer-1 at 5 µM. No statistical methods were used to pre-determine sample sizes, but our sample sizes are similar to those reported in previous publications. Data collection and analysis were not performed blind, but all samples were processed and analysed under identical conditions. For more details, refer to our protocol^55^.

### In vitro erythroid differentiation

At 48 h post-thawing, mPB-derived HSPCs were electroporated with Cas9 RNPs targeting the *BCL11A +*58 enhancer^6,47,56^ and were immediately transferred into erythroid differentiation medium^48^. The differentiation medium consisted of IMDM (Life Technologies) supplemented with 2% human AB plasma (SeraCare), 3% human AB serum (Atlanta Biologicals), 1% penicillin/streptomycin (Life Technologies), 3 U ml^−1^ heparin (Hospira) and 10 μg ml^−1^ recombinant human insulin (Lilly). During phase I of differentiation (days 0–6), the medium was further supplemented with 200 μg ml^−1^ holo-human transferrin (Sigma), 1 ng ml^−1^ of recombinant human IL-3 (Peprotech), 10 ng ml^−1^ human SCF (Peprotech) and 3 U ml^−1^ erythropoietin (Amgen). In phase II (days 7–11), IL-3 was removed from the medium. During phase III (days 12–21), both IL-3 and SCF were omitted, and the holo-transferrin concentration was increased to 1 mg ml^−1^.

### Xenotransplantation and animal models

All animal procedures were performed under protocols approved by the Institutional Animal Care and Use Committee of Boston Children’s Hospital (protocol no. 2257). CD34⁺ cells derived from CB were cultured in cytokine-free conditions and after 7 days of expansion in culture, 20,000 input cells (yielding approximately 50,000 total cells) were injected per mouse via the tail vein into NBSGW immunodeficient and *Kit* mutant mice (JAX#026622). For secondary transplants, CD34⁺ cells purified from the BM of primary recipients were transplanted using two different approaches: (1) a one-donor–one-recipient strategy, in which all CD34⁺ cells from an individual primary mouse were transplanted into a single secondary recipient and (2) a pooled strategy, in which CD34⁺ cells from multiple primary mice within the same experimental group were pooled, and equal numbers of cells were transplanted into separate secondary recipients. mPB-derived CD34⁺ cells were cultured in serum-free conditions and after 4 days of expansion in culture, 150,000 input cells (yielding approximately 1,400,000 total cells) were injected per mouse via the tail vein into NBSGW mice.

Mice were randomly assigned to treatment or control groups at the time of transplantation to minimize bias. No specific method of randomization was applied, but animals were distributed across groups to ensure comparable sex and age. Data collection and analysis were not performed blind, but all animals were processed and analysed under identical conditions. For these reasons, no data points were excluded from the analyses.

To prevent infections, the mice were provided with autoclaved sulfatrim antibiotic water, which was changed weekly. To monitor engraftment, peripheral blood was collected at 4, 8, 12 and 16 weeks post-transplantation through retro-orbital sampling. At 12 or 16 weeks post-transplantation, the animals were euthanized, and their BMs and spleens were collected for analysis. BM cells were obtained by flushing the bilateral femurs and tibias, hips, and sternum, while spleens were carefully minced. Human chimerism in the collected samples was assessed by flow cytometry using anti-human CD45 and anti-mouse CD45 antibodies. The composition of specific cell lineages in the organs was evaluated using lineage-specific markers: hCD3 (T cells), hCD33 (myeloid cells) and CD19 (B cells).

### C.f.u. cell assay

The colony-forming unit (c.f.u.) cell assay was performed using CD34^+^ cells derived from the BM of mice transplanted with CB- or mPB-derived cells, cultured either in the presence or absence of Lip-1. A total of 2,500 cells were plated in a methylcellulose-based medium (MethoCult H4434, StemCell Technologies) that contains the cytokines IL-3, stem cell factor (SCF), granulocyte–macrophage colony-stimulating factor (GM-CSF) and erythropoietin (EPO) and which is supplemented with 100 IU ml^−1^ penicillin and 100 µg ml^−1^ streptomycin. At 2 weeks post-plating, colonies were counted in a blinded fashion, and erythroid, myeloid and mixed colonies were identified according to morphological criteria.

### CRISPR–Cas9 RNP nucleofection

The Cas9 RNP complexes were assembled by combining 2.1 µl of DPBS, 1.2 µl of 100 mM sgRNA in IDTE pH 7.5 (IDT) and 1.7 µl of 62 mM Alt-R S.p. HiFi Cas9 Nuclease V3 (IDT, 1081061) and incubating at room temperature for 10–30 min. The assembled RNP complex was then mixed with Lonza P3 primary cell nucleofection reagent (Lonza, V4XP-3032) in the presence of 1 µl of 100 mM stock of IDT nucleofection enhancer. The mixture was delivered into CD34^+^ HSPCs by nucleofection using the Lonza 4D nucleofector system with the EO100 program 2 or 7 days after thawing in serum- or cytokine-free culture medium, respectively. The cells were collected for genomic DNA extraction at least 72 h post-nucleofection and PCR fragments flanking the editing site (at least 250 bp upstream and downstream) were amplified and sent for Sanger sequencing to assess editing efficiencies. Sanger traces were imported to TIDE CRISPR version 3.2.0 for indel measurement with 100 bp left boundary and automatically set at break site −10 bp as alignment window, 115–515 bp decomposition window, 40 bp indel size range and 0.05 *P* value. Sequences of all gRNAs and primers used in this study are provided in the Supplementary Table 3.

### Base editor protein electroporation

RNP complexes were assembled by combining 2.1 µl of DPBS, 1.57 µl of chemically modified sgRNAs (IDT) resuspended at 100 mM in IDTE pH 7.5 (IDT, 11-01-02-02) and the ABE8e protein, and incubating at room temperature for 10–30 min. ABE8e protein was purified as previously described^44^ while the final amount of base editor protein per electroporation ranged between 20 and 40 µg and was optimized using the base editing activity of the batch as assessed by titration experiments in primary HSPCs. The mixture was delivered into CD34^+^ HSPCs by nucleofection using the Lonza 4D nucleofector system with the EO100 program. The cells were collected, and genomic DNA was extracted at least 72 h post-nucleofection for next-generation sequencing-based calculation of editing efficiency.

### Real-time PCR analysis

The total RNA was obtained using the Quick-DNA/RNA Microprep Plus (Zymo research) purification kit according to the manufacturer’s instructions. Then, 100–500 ng of total RNA was used for reverse transcription using the iScript cDNA synthesis kit (Bio-Rad). The cDNA product was used for real-time PCR analysis using iQ SYBR green supermix (Bio-Rad). Three technical replicates were performed for each sample, and the mean value was selected for further comparisons. The relative expression of each target gene was first normalized to *ACTB* housekeeping gene expression and then represented as the fold change (2^−^^ΔΔ*Ct*^) relative to the indicated control condition. Sequences of all primers used in this study are provided in the Supplementary Table 3.

### Immunophenotypic and apoptosis analysis

For immunophenotypic analyses (performed on LSRII or LSRFortessa; BD Pharmingen) of ex vivo cultured HSPCs, the cellular suspension (up to 1 × 10^6^ cells) was incubated for 30 min with different fluorescent-labelled antibodies: 1:100 dilution of anti-human CD34 APC-Cy7 (BioLegend, 343614), 1:50 dilution of anti-human CD133 Super Bright 436 (Invitrogen, 62-1338-42), 1:100 dilution of anti-human CD90 PE-Cy7 (BD Biosciences, 561558), 1:50 dilution of anti-human CD45RA AlexaFluor-700 (BioLegend, 304120), 1:100 dilution of anti-human CD201 (EPCR) PE (BioLegend, 351904) and 1:40 dilution of anti-human CD49c (ITGA3) APC (BioLegend, 343808). Immunophenotypic staining was combined with 1:200 dilution of Apotracker Green (BioLegend, 427402) viability staining according to the manufacturer’s instructions.

For erythroid differentiation analysis, cells at the indicated stage of differentiation were collected and incubated with the following fluorescent-labelled antibodies: 1:150 dilution of anti-human CD235a APC-Cy7 (BioLegend, 349116) and 1:150 dilution of anti-human CD71 BV421 (BioLegend, 334122). Dead cells were excluded according to their positivity to Apotracker Green (BioLegend, 427402) staining.

For immunophenotypic analyses (performed on LSRII or LSRFortessa; BD Pharmingen) of cells retrieved from mouse organs, cells were stained with different fluorescent-labelled antibodies, for peripheral blood and spleen samples: 1:200 dilution of Apotracker Green (BioLegend, 427402), 1:100 dilution of anti-human CD45 APC (BioLegend, 304012), 1:50 dilution of anti-mouse CD45 PE (BioLegend, 103106), 1:100 dilution of anti-human CD19 APC-Cy7 (BD Biosciences, 560727), 1:100 dilution of anti-human CD33 BV421 (BioLegend, 303416) and 1:100 dilution of anti-human CD3 BV605 (BD Biosciences, 563217); for BM samples: 1:200 dilution of Apotracker Green (BioLegend, 427402), 1:100 dilution of anti-human CD45 APC (BioLegend, 304012), 1:50 dilution of anti-mouse CD45 PE (BioLegend, 103106), 1:100 dilution of anti-human CD19 APC-Cy7 (BD Biosciences, 560727), 1:100 dilution of anti-human CD34 BV421 (BioLegend, 343610), 1:100 dilution of anti-human CD90 PE-Cy7 (BD Biosciences, 561558) and 1:50 dilution of anti-human CD45RA AlexaFluor-700 (BioLegend, 304120). Single-stained cells were used as controls and Rainbow Calibration Particles (Invitrogen, A34305) were used to calibrate the instrument. Data were analysed using the FlowJo software.

### Western blot

For immunoblot analysis, HSPCs were cultured in serum-free expansion medium for 4 days in the presence or absence of sodium selenite. Viable cells (negative to Apotracker) were sorted with a 100 µm nozzle on a BD FACSAria Fusion (BD Biosciences), according to the surface expression of CD34, CD45RA and CD90 markers. Sorted CD34^+^CD45RA^−^CD90^+^ cells were washed twice with cold PBS and lysed on ice for 30 min in RIPA buffer (Thermo Fisher Scientific, 89900), supplemented with protease and phosphatase inhibitor mini tablets (Thermo Fisher Scientific, A32965). Lysates were centrifuged at 16,000*g* for 10 min at 4 °C, and the protein concentration in the supernatants was quantified using the Pierce BCA Protein Assay Kit (Thermo Fisher Scientific, 23225), according to the manufacturer’s instructions. Equal amounts of protein were separated on 10% Mini-PROTEAN TGX Gels (Bio-Rad, 4561036) and transferred onto PVDF membranes (Millipore, IPVH00010) using the Trans-Blot Turbo Transfer System (Bio-Rad). Membranes were blocked in Intercept blocking buffer (LICORbio, 927-70001) and incubated overnight at 4 °C with primary antibodies against GPX4 (CellSignaling Technology, 52455; 1:1,000) and Actin (Santa Cruz, sc-8432; 1:1,000). After washing, membranes were incubated for 1 h at room temperature with HRP-conjugated anti-rabbit or anti-mouse secondary antibodies (Bio-Rad, 1706515 and 1706516, respectively; 1:5,000). Signal detection was performed using Clarity-Western ECL Substrate (Bio-Rad, 170-5061), and images were acquired using a ChemiDoc Imaging System (Bio-Rad).

### GSH measurement

Total GSH levels were measured using the GSH-Glo Glutathione Assay (Promega, V6911) according to the manufacturer’s instructions. In brief, HSPCs were cultured in serum-free expansion medium for 4 days in the presence or absence of β-ME. Viable cells (negative to Apotracker) were sorted with a 100 µm nozzle on a BD FACSAria Fusion (BD Biosciences), according to the surface expression of CD34, CD45RA and CD90 markers. Sorted CD34^+^CD45RA^−^CD90^+^ cells were resuspended in 50 µl of PBS and plated in white 96-well plates (Thermo Fisher Scientific, 15042) at a density of 10,000 cells per well in technical triplicates. An equal volume of GSH-Glo Reagent, containing a luciferin derivative and GSH *S*-transferase, was added to each well and plates were incubated for 30 min at room temperature to allow conversion of the substrate in the presence of intracellular GSH. After this incubation, Luciferin Detection Reagent was added, and following a 15-min incubation at room temperature, luminescence was measured using a microplate luminometer (CLARIOstar). GSH concentrations were determined by comparing luminescence values with a standard curve generated using known concentrations of GSH.

### Protein synthesis measurement

Protein synthesis rates were assessed using OP-puro (Cayman Chemical, 601100) incorporation. HSPCs were cultured for 4 days in serum-free expansion medium in the presence or absence of Lip-1. On the day of analysis, OP-puro (1:400 dilution from stock) was added to the culture medium and cells were incubated at 37 °C for 2 h. After incubation, cells were washed with PBS and fixed using Cell-Based Assay Fixative for 5 min at room temperature. To detect OP-puro incorporation, cells were resuspended in FAM-Azide staining solution and incubated for 30 min at room temperature in the dark. Following staining, cells were washed with Cell-Based Assay Wash Buffer and analysed using an LSRFortessa flow cytometer (BD Biosciences). As a negative control, cells were pretreated with cycloheximide (50 µg ml^−1^) for 1 h before OP-puro addition to inhibit protein synthesis. Data were analysed with FlowJo software, and the mean fluorescence intensity was used to quantify relative translation activity.

### Fetal haemoglobin detection in cells

Erythroid differentiation phase III cellular suspension of approximately 3–5 × 10⁴ cells was washed with 3 ml of 2% FBS–DPBS and fixed with 4% paraformaldehyde (Santa Cruz Biotechnology) at room temperature for 15 min. Following fixation, the cells were washed again with 3 ml of 2% FBS–DPBS and then permeabilized with 0.2% tween 20 in PBS for 5 min. Subsequently, the cells were stained with a 1:200 dilution of anti-human HbF PE fluorescent antibody (BD Biosciences, 560041) for 30 min at room temperature. After a final wash with 2% FBS in PBS, sample acquisition was performed using an LSRFortessa flow cytometer. The collected data were then analysed using FlowJo software.

### Cell proliferation

After thawing, HSPCs were stained with Cell Trace CFSE (Thermo Scientific, 50-591-407) according to the manufacturer’s instructions. The cells were resuspended in 1× DPBS at a concentration of 10^6^ cells ml^−1^. Then, 1 µl of Cell Trace solution was added per ml of cell suspension, resulting in a final concentration of 5 µM. Cells were incubated for 20 min at 37 °C, protected from light. Following this, cells were incubated with five times the original staining volume of 1× DPBS plus 2% FBS for 5 min. After centrifugation, the cells were resuspended in the appropriate culture medium volume and incubated for at least 30 min before analysis. Sample acquisition was performed on an LSRFortessa flow cytometer (BD Pharmingen) and the collected data were analysed using FlowJo software.

### Lipid peroxidation analysis

HSPCs were initially stained with anti-human CD34 BV421 (BioLegend, 343610), anti-human CD90 PE-Cy7 (BD Biosciences, 561558) and anti-human CD45RA AlexaFluor-700 (BioLegend, 304120), as previously described. Following this, the cells were incubated with the BODIPY C11 lipid probe (Invitrogen) according to the manufacturer’s instructions. In brief, cells were stained with 5 µM BODIPY 581/591 C11 reagent in PBS at 37 °C for 30 min. After staining, the labelled cells were washed and analysed using an LSRFortessa flow cytometer (BD Pharmingen, D3861).

The lipid peroxidation state of each group was quantified by calculating the ratio of the mean fluorescence intensity of oxidized lipids (detected as green signal) to that of reduced lipids (detected as the red signal).

### Reversed-phase C8 chromatography–positive-ion mode MS detection to measure lipids

Analyses of polar and non-polar lipids were conducted using an liquid chromatography–mass spectrometry (LC–MS) system comprising a Shimadzu Nexera X2 U-HPLC (Shimadzu Corp.) coupled to an Exactive Plus orbitrap mass spectrometer (Thermo Fisher Scientific). HSPCs were cultured in chemically defined cytokine-free conditions^12^ for 10 days in the presence or absence of Lip-1. Viable cells (negative to Apotracker) were sorted with a 100 µm nozzle on a BD FACSAria Fusion (BD Biosciences), according to the surface expression of CD34, CD45RA and CD90 markers. Then 5 × 10^5^ sorted CD34^+^CD45RA^−^CD90^+^ and CD34^+^CD45RA^−^CD90^−^ cells were collected in 1.5 ml Eppendorf tubes containing 100 μl of isopropanol. Samples were centrifuged at 10,000*g* for 10 min and 10 µl of supernatant was injected directly onto a 100 × 2.1 mm, 1.7 µm ACQUITY BEH C8 column (Waters). The column was eluted isocratically with 80% mobile phase A (95:5:0.1 vol/vol/vol 10 mM ammonium acetate/methanol/formic acid) for 1 min followed by a linear gradient to 80% mobile-phase B (99.9:0.1 vol/vol methanol/formic acid) over 2 min, a linear gradient to 100% mobile phase B over 7 min and then 3 min at 100% mobile-phase B. MS analyses were carried out using electrospray ionization in the positive ion mode using full scan analysis over 220–1,100 *m*/*z* at 70,000 resolution and 3 Hz data acquisition rate. Other MS settings were sheath gas 50, in source CID 5 eV, sweep gas 5, spray voltage 3 kV, capillary temperature 300 °C, S-lens RF 60, heater temperature 300 °C, microscans 1, automatic gain control target 1e6 and maximum ion time 100 ms. Raw data were processed using TraceFinder software (Thermo Fisher Scientific) for targeted peak integration and a manual review of a subset of identified lipids and using Progenesis QI (Nonlinear Dynamics) for peak detection and integration of both lipids of known identify and unknowns. Lipid identities were determined based on comparison to reference plasma extracts and are denoted by total number of carbons in the lipid acyl chain(s) and total number of double bonds in the lipid acyl chain(s).

### Clonal haematopoiesis mutation amplicon sequencing and data analysis

HSPCs derived from four different donors were cultured in chemically defined cytokine-free conditions^12^ for up to 3 weeks in the presence or absence of Lip-1. To assess the potential acquisition of a clonal haematopoiesis and indeterminate potential (CHIP) mutation, an early (7–10 days) and a late (17–25 days) sample from the same cultures were analysed by deep amplicon sequencing (Supplementary Table 2). The presence of mutations in donor-derived cultures was assessed using a targeted gene panel that has been previously described^32^. The panel consists of 137 amplicons across 24 genes, covering >95% of observed CHIP mutations. One sample (#14) failed sequencing for technical reasons, while for all the others paired-end sequencing yielded 11^6^–25^6^ reads per sample. Each sample was processed by an in-house variant calling pipeline. Specifically, sequencing adaptors were trimmed using cutadapt^57^ and assessed in FastQC (https://www.bioinformatics.babraham.ac.uk/projects/fastqc/). Trimmed reads were mapped to hg38 using BWA mem (http://arxiv.org/abs/1303.3997). We then marked duplicates using Picard (http://broadinstitute.github.io/picard/), recalibrated base qualities using GATK^58^, and ran Mutect2^59^ with default parameters to call somatic variants. The produced VCF file (unfiltered Mutect2 results) was filtered by FilterMutectCalls (with -max-events-in-region 3) and BCFtools^59,60^, to select variants with sequencing depth >= 50, >= 5 reads supporting the variant, and allele frequency >= 0.01. In addition, we separately assessed the commonly artifactual *ASXL1* c.1934dupG frameshift with allele frequency >0.05, which often fails the ‘slippage’ filter of Mutect2 but was reported as a true variation in some samples^61^. Called variants were then annotated using the Ensembl Variant Effect Predictor^62^ and manually inspected in the Interactive Genome Viewer^63^ to remove germline variants and false positive calls with low support or polymerase slippage. In addition, we conducted a cross-abundance analysis to assess the number of occurrences of each selected variant in unfiltered Mutect2 results across all samples in this sequencing run, including samples unrelated to this project. Three variants were recurrent (Extended Data Fig. 5). One of them, a missense mutation in *TP53* with a variant allele frequency (VAF) close to 0.5, is a germline variant, being present in all four samples from donor CB-52 (Supplementary Table 2). This specific variant (NM_000546.6(TP53):c.869 G > A (p.Arg290His)) has been annotated by the ClinGen TP53 Variant Curation Expert Panel and other expert panels to be a benign polymorphism found in the population^64^. The two other variants are single-base insertions in homopolymeric tracts within *ASXL1* and *IDH2* genes; we consider them PCR artefacts as they are present across multiple independent samples from this study and healthy donor BM samples from an independent study (Extended Data Fig. 5, grey bars). Of three additional variants that were reported, two were found to be artefacts upon manual inspection because sequencing reads supporting these variants were also present in several unrelated samples, and one was a synonymous variant in *ZBTB33* with VAF <2%, supported by less than 10 reads. Overall, the targeted sequencing of 137 amplicons did not show any reliable CHIP-associated mutations at a VAF threshold ≥1%.

### scRNA-seq and analysis

Droplet-based digital 3′-end scRNA-seq was performed on a Chromium Single-Cell Controller (10X Genomics) using the Chromium Next GEM Single Cell 3′ Reagent Kit v3.1 according to the manufacturer’s instructions. CB HSPCs derived from two different donors were cultured in chemically defined cytokine-free conditions^12^ for 10 days in the presence or absence of Lip-1. Viable cells (negative to Apotracker) were sorted with a 100 µm nozzle on a BD FACSAria Fusion (BD Biosciences), according to the surface expression of CD34^+^CD45RA^−^CD90^+^ markers. Sorted cells were collected in 1.5 ml Eppendorf tubes containing 100 μl of 1× DPBS. The sorted cells were counted with Trypan Blue solution 0.4% (GIBCO) and roughly 2 × 10^4^ viable cells from each sample were utilized for the subsequent procedure (estimated recovery: 10^4^ cells per sample). Briefly, single cells were partitioned in Gel Beads in Emulsion and lysed, followed by RNA barcoding, reverse transcription and PCR amplification (11 cycles). scRNA-seq libraries were prepared according to the manufacturer’s instructions, checked and quantified on LabChip GX Touch HT (Perkin Elmer) and Qubit 3.0 (Invitrogen) instruments. Sequencing was performed on a Nova Seq S2 (Illumina). The raw scRNA-seq FASTQ files were processed with the CellRanger (v8.0.1) pipeline to map in the reference genome (GRCh38). We excluded cells with unique molecular identifier (UMI) counts less than 1,000 or mitochondrial UMI fraction higher than 20%, and removed potential doublets by a threshold of doublet score >0.2 using ScrubletR, which resulted in a total of 29,096 cells for the Lip-1 (replicate 1 = 5,991 and replicate 2 = 4,935) and untreated (replicate 1 = 8,991 and replicate 2 = 9,179) groups (Supplementary Table 1). The symphony R package^65^ was used to project the cells on the human BM^30^ (https://github.com/andygxzeng/BoneMarrowMap), and the scRNA-seq reference built from 10 day-expanded CD34^+^ cells in cytokine-free medium, and the predicted cell type was further curated to match the 12 haematopoietic cell types presented in ref. ^12^. A standard Seurat framework (v4.4.0) was used to conduct normalization, principal component analysis (PCA) and dimensionality reduction. The feature-barcode matrix was normalized by the total read count and log-transformed, and the top 3,000 variable features were selected by the vst method in the FindVariableFeatures function. The normalized expression was scaled by Seurat’s ScaleData function and PCA was performed using the RunPCA function (npc = 30). The sample-dependent technical variation was corrected by using Harmony^66^. Uniform Manifold Approximation and Projection (UMAP) was conducted to reduce dimensions to embed the cells into two-dimensional space. The HSC signature score was measured by applying AUCell^67^ using CD34 and HLF RNA expression. Seurat’s FindMarkers function using the wilcox method was applied to the HSC compartment to identify differentially expressed genes between Lip-1 and untreated cells with a significance threshold of Benjamini–Hochberg-adjusted *P* < 0.05, log_2_-transformed fold change >0.1, and minimum per cent of expressed cells >10%. Gene set enrichment analysis was performed using the fGSEA package (https://github.com/ctlab/fgsea/) using GO Biological Process 2021 database. Profiling of chromosome copy numbers was assessed by Numbat^33^ (v1.4.0), run using default parameters. The Sakurai et al.^12^ dataset was used as the expression reference for all samples. Figures were generated using R (v4.4).

### Statistics and reproducibility

In all experiments, data are presented as mean ± s.e.m. Data normality and homogeneity of variances were formally assessed using the Shapiro–Wilk test. The appropriate statistical test was then selected based on these results: when *P* > 0.05 (indicating a normal distribution), a two-tailed Student’s *t*-test was applied; when *P* < 0.05 (non-normal distribution), a two-tailed non-parametric Mann–Whitney test was used instead. When one of the two samples was a default value (as in fold change comparison), the one-sample *t*-test and Wilcoxon test was applied. When comparing three or more samples, Levene’s test was first used to test equality of variance. If the variance across all samples tested did not significantly differ, one-way or two-way ANOVA with Dunnett’s test (for multiple comparisons where no reference group is defined) or Tukey’s test (for multiple comparisons where reference group is defined) a post hoc analysis was used. If the variance across samples was tested to be significantly different, the Kruskal–Wallis test was used instead of ANOVA, with the Dunn test as the post hoc multiple comparison test. To account for interdonor variability in in vivo experiments, a linear mixed-effects model was applied, with Lip-1 treatment as a fixed effect and donor as a random effect. Model fitting was performed using restricted maximum likelihood estimation via the lme4 R package. Statistical significance of fixed effects was assessed using *t*-tests with Satterthwaite’s approximation for degrees of freedom, implemented through the lmerTest R package. All statistical tests were performed in Graphpad software or R when statistical tests were not available through Graphpad.

### Reporting summary

Further information on research design is available in the Nature Portfolio Reporting Summary linked to this article.

## Online content

Any methods, additional references, Nature Portfolio reporting summaries, source data, extended data, supplementary information, acknowledgements, peer review information; details of author contributions and competing interests; and statements of data and code availability are available at 10.1038/s41556-025-01814-7.

## Supplementary information

Reporting Summary

Supplementary Table 1 scRNA-seq data.

Supplementary Table 2 List of samples sequenced for clonal hematopoiesis mutation amplicons.

Supplementary Table 3 Sequences of all gRNAs and primers used in this study.

## Source data

Source Data Fig. 1 Statistical source data.

Source Data Fig. 2 Statistical source data.

Source Data Fig. 3 Statistical source data.

Source Data Fig. 4 Statistical source data.

Source Data Fig. 5 Statistical source data.

Source Data Fig. 6 Statistical source data.

Source Data Extended Data Fig./Table 2 Statistical source data and unprocessed blots of data used in Extended Data Fig. 2k.

Source Data Extended Data Fig./Table 2 Statistical source data and unprocessed blots of data used in Extended Data Fig. 2k.

Source Data Extended Data Fig./Table 3 Statistical source data.

Source Data Extended Data Fig./Table 5 Statistical source data.

Source Data Extended Data Fig./Table 8 Statistical source data.

Source Data Extended Data Fig./Table 9 Statistical source data.

Source Data Extended Data Fig./Table 10 Statistical source data.

## Data Availability

Sequencing data that support the findings of this study have been deposited in the Gene Expression Omnibus (GEO) under accession code GSE276160. Previously published scRNA-seq of 10 day-expanded CD34^+^ cells data from Sakurai et al. re-analysed here are available under accession code GSE192519. All other data supporting the findings of this study are available from the corresponding author on reasonable request. Source data are provided with this paper.
