# Supplementary material for: Inhibiting ferroptosis enhances ex vivo expansion of human haematopoietic stem cells
Source: Nat Cell Biol. 2025 Nov 18;27(12):2214–24. doi: 10.1038/s41556-025-01814-7 (PMC12680438; doi:10.1038/s41556-025-01814-7)
Supplement: Supplementary file 1 — Reporting Summary [file 41556_2025_1814_MOESM1_ESM.pdf]

Corresponding author(s): Vijay G. Sankaran

Last updated by author(s): Oct 14, 2025

Reporting Summary

Nature Portfolio wishes to improve the reproducibility of the work that we publish. This form provides structure for consistency and transparency in reporting. For further information on Nature Portfolio policies, see our Editorial Policies and the Editorial Policy Checklist.

Statistics

For all statistical analyses, confirm that the following items are present in the figure legend, table legend, main text, or Methods section.

n/a	Confirmed	
☐	☒	The exact sample size (*n*) for each experimental group/condition, given as a discrete number and unit of measurement
☐	☒	A statement on whether measurements were taken from distinct samples or whether the same sample was measured repeatedly
☐	☒	The statistical test(s) used AND whether they are one- or two-sided<br>*Only common tests should be described solely by name; describe more complex techniques in the Methods section.*
☒	☐	A description of all covariates tested
☐	☒	A description of any assumptions or corrections, such as tests of normality and adjustment for multiple comparisons
☐	☒	A full description of the statistical parameters including central tendency (e.g. means) or other basic estimates (e.g. regression coefficient) AND variation (e.g. standard deviation) or associated estimates of uncertainty (e.g. confidence intervals)
☐	☒	For null hypothesis testing, the test statistic (e.g. *F*, *t*, *r*) with confidence intervals, effect sizes, degrees of freedom and *P* value noted<br>*Give P values as exact values whenever suitable.*
☒	☐	For Bayesian analysis, information on the choice of priors and Markov chain Monte Carlo settings
☒	☐	For hierarchical and complex designs, identification of the appropriate level for tests and full reporting of outcomes
☒	☐	Estimates of effect sizes (e.g. Cohen's *d*, Pearson's *r*), indicating how they were calculated

Our web collection on statistics for biologists contains articles on many of the points above.

Software and code

Policy information about availability of computer code

Data collection	Flow cytometry analyses were performed on LSRII or LSRFortessa (BD Pharmingen) using BDFACS Diva software. Cell sorting was performed on a BD FACS S6 Symphony (BD Biosciences) using BDFACS Diva software.<br>Real-time qPCR was performed by CFX96 Real-Time System (Bio-Rad).<br>scRNA-Seq libraries were quantified on Bioanalyzer High Sensitivity DNA (Agilent) and Qubit 3.0 (Invitrogen) instruments.<br>High-throughput sequencing was performed on NovaSeq S2 (100 Cycles) (Illumina).<br>Luminescence was measured by CLARIOstar (BMG LABTECH)
Data analysis	GraphPad Prism (v10) was used to plot data and perform statistical analyses.<br>Flow Cytometry data were analyzed with FlowJo, BD (v10).<br>Gene editing efficiency was measured using TIDE platform (https://tide.nki.nl/)<br>Western blot analysis was performed using ImageJ.

For manuscripts utilizing custom algorithms or software that are central to the research but not yet described in published literature, software must be made available to editors and reviewers. We strongly encourage code deposition in a community repository (e.g. GitHub). See the Nature Portfolio guidelines for submitting code & software for further information.

Data

Policy information about availability of data

All manuscripts must include a data availability statement. This statement should provide the following information, where applicable:

- Accession codes, unique identifiers, or web links for publicly available datasets
- A description of any restrictions on data availability
- For clinical datasets or third party data, please ensure that the statement adheres to our policy

Sequencing data that support the findings of this study have been deposited in the Gene Expression Omnibus (GEO) under accession code GSE276160.

Research involving human participants, their data, or biological material

Policy information about studies with human participants or human data. See also policy information about sex, gender (identity/presentation), and sexual orientation and race, ethnicity and racism.

Reporting on sex and gender	We worked on purified primary cells from both female and male healthy donors. No statistical methods were used to pre-determine sample sizes, but our sample sizes are similar to those reported in previous publications.
Reporting on race, ethnicity, or other socially relevant groupings	We worked on purified primary cells for which information on race, ethnicity, or other socially relevant groupings were not taken into account in the design of this study.
Population characteristics	The experiments were performed on cells from healthy donors.
Recruitment	Human CD34+ HSPCs from mobilized peripheral blood of healthy adults were obtained from the Cooperative Center of Excellence in Hematology at the Fred Hutchinson Cancer Research Center.<br>Human CD34+ HSPCs were also sourced from cord blood, obtained from the Dana-Farber Cancer Institute or Brigham and Women's Hospital as discarded deidentified samples.
Ethics oversight	All experiments were conducted in accordance with relevant ethical regulations. The use of human hematopoietic stem and progenitor cells (HSPCs) from cord blood (CB) and mobilized peripheral blood (mPB) was approved by the Institutional Review Board of Boston Children's Hospital (protocol IRB-P00048735), and informed consent was obtained from all donors in accordance with the Declaration of Helsinki. Donors did not receive any form of financial or material compensation for their participation.

Note that full information on the approval of the study protocol must also be provided in the manuscript.

Field-specific reporting

Please select the one below that is the best fit for your research. If you are not sure, read the appropriate sections before making your selection.

☒ Life sciences ☐ Behavioural & social sciences ☐ Ecological, evolutionary & environmental sciences

For a reference copy of the document with all sections, see nature.com/documents/nr-reporting-summary-flat.pdf

Life sciences study design

All studies must disclose on these points even when the disclosure is negative.

Sample size	No statistical methods were used to pre-determine sample sizes. The sample size for each experiment was determined by the total number of available cells, which is constrained by the human source of the material, to be split among each experimental condition. Whenever possible we aimed to reach at least 3 replicates per group, thus reaching a minimum and sensible operational criteria for carrying out at least nonparametric statistics.
Data exclusions	No data were excluded from the analysis.
Replication	Most experiments were repeated more than three times. The number of biological replicates is specified for each experiment in figure legends. All attempts at replication were successful.
Randomization	Mice were randomly distributed to each experimental group.
Blinding	Data collection and analysis were not performed blind, but all samples were processed and analyzed under identical conditions.

Reporting for specific materials, systems and methods

We require information from authors about some types of materials, experimental systems and methods used in many studies. Here, indicate whether each material, system or method listed is relevant to your study. If you are not sure if a list item applies to your research, read the appropriate section before selecting a response.

Materials & experimental systems

n/a	Involved in the study
☐	☒ Antibodies
☒	☐ Eukaryotic cell lines
☒	☐ Palaeontology and archaeology
☐	☒ Animals and other organisms
☒	☐ Clinical data
☒	☐ Dual use research of concern
☒	☐ Plants

Methods

n/a	Involved in the study
☒	☐ ChIP-seq
☐	☒ Flow cytometry
☒	☐ MRI-based neuroimaging

Antibodies

Antibodies used

*Apotracker Green (Supplier: BioLegend; Catalog n°427402; Dilution: 1:200)
*CD34 APC/Cyanine7 anti-human (Supplier: BioLegend; Catalog n°343614; Clone: 561; Dilution: 1:100)
*CD133 (Prominin-1) Super Bright 436, anti-human (Supplier: Invitrogen; Catalog n°62-1338-42; Clone: TMP4; Dilution: 1:50)
*CD90 PE-Cy7, anti-human (Supplier: BD Biosciences; Catalog n° 561558; Clone: 5E10; Dilution: 1:100)
*CD45RA Alexa Fluor 700, anti-human (Supplier: BioLegend; Catalog n° 304120; Clone: HI100; Dilution: 1:50)
*CD201 (EPCR) PE, anti-human (Supplier: BioLegend; Catalog n° 351904; Clone: RCR-401; Dilution: 1:100)
*CD49c (integrin α3) APC, anti-human (Supplier: BioLegend; Catalog n° 343808; Clone: ASC-1; Dilution: 1:40)
*CD34 Brilliant Violet 421, anti-human (Supplier: BioLegend; Catalog n° 343610; Clone: 561; Dilution: 1:100)
*CD90 APC, anti-human (Supplier: BD Biosciences; Catalog n°561971; Clone: 5E10; Dilution: 1:100)
*CD45RA APC/Cyanine7, anti-human (Supplier: BioLegend; Catalog n°304128; Clone: HI100; Dilution: 1:50)
*CD45 APC, anti-human (Supplier: BioLegend; Catalog n°304012; Clone: HI30; Dilution: 1:100)
*CD45 PE, anti-mouse (Supplier: BioLegend; Catalog n°103106; Clone: 30-F11; Dilution: 1:50)
*CD19 APC-H7, anti-human (Supplier: BD Biosciences; Catalog n° 560727; Clone: HIB19; Dilution: 1:100)
*CD33 Brilliant Violent 421, anti-human (Supplier: BioLegend; Catalog n°303416; Clone: WM53; Dilution: 1:100)
*CD3 BV605 anti-human (Supplier: BD Biosciences; Catalog n°563217; Clone: SK7; Dilution: 1:100)
*CD235a (Glycophorin A)-APC/Cyanine7, anti-human (Supplier: BioLegend; Catalog n° 349116; Clone: HI264; Dilution: 1:100)
*CD71 Brilliant Violet 421, anti-human (Supplier: BioLegend; Catalog n°334122; Clone: CY1G4; Dilution: 1:100)
*HBF (Human Fetal Hemoglobin) PE, anti-human (Supplier: BD Biosciences; Catalog n°560041; Clone: 2D12; Dilution: 1:100)
*Actin (Supplier: Santa Cruz; Catalog n° sc-8432; Dilution: 1:1000)
*GPX4 (Supplier: CellSignaling Technology; Catalog n° 52455; Dilution: 1:1000)

Validation

*https://d1spbj2x7qk4bg.cloudfront.net/ja-jp/products/apotracker-green-18527?displayInline=true&filename=Apotracker%E2%84%A2%20Green.pdf&leftRightMargin=15&pdf=true&topBottomMargin=15&v=20241112062843
*https://d1spbj2x7qk4bg.cloudfront.net/en-gb/products/apc-cyanine7-anti-human-cd34-antibody-12973?displayInline=true&filename=APC/Cyanine7%20anti-human%20CD34%20Antibody.pdf&leftRightMargin=15&pdf=true&topBottomMargin=15&v=20241107063036
*https://www.thermofisher.com/order/genome-database/dataSheetPdf?producttype=antibody&productsubtype=antibody_primary&productId=62-1338-42&version=Local
*https://www.bdbiosciences.com/content/dam/bdb/products/global/reagents/flow-cytometry-reagents/research-reagents/single-color-antibodies-ruo/561xxx/5615xx/561558_base/pdf/561558.pdf
*https://d1spbj2x7qk4bg.cloudfront.net/de-at/products/alexa-fluor-700-anti-human-cd45ra-antibody-3421?displayInline=true&filename=Alexa%20Fluor%C2%AE%20700%20anti-human%20CD45RA%20Antibody.pdf&leftRightMargin=15&pdf=true&topBottomMargin=15&v=20241102123737
*https://d1spbj2x7qk4bg.cloudfront.net/en-gb/products/pe-anti-human-cd201-epcr-antibody-7240?displayInline=true&filename=PE%20anti-human%20CD201%20(EPCR)%20Antibody.pdf&leftRightMargin=15&pdf=true&topBottomMargin=15&v=20241102123737
*https://d1spbj2x7qk4bg.cloudfront.net/en-ie/products/apc-anti-human-cd49c-integrin-alpha3-antibody-6810?displayInline=true&filename=APC%20anti-human%20CD49c%20(integrin%CE%B13)%20Antibody.pdf&leftRightMargin=15&pdf=true&topBottomMargin=15&v=20241002064001
*https://d1spbj2x7qk4bg.cloudfront.net/de-at/products/brilliant-violet-421-anti-human-cd34-antibody-7708?displayInline=true&filename=Brilliant%20Violet%20421%E2%84%A2%20anti-human%20CD34%20Antibody.pdf&leftRightMargin=15&pdf=true&topBottomMargin=15&v=20241002064001
*https://www.bdbiosciences.com/content/dam/bdb/products/global/reagents/flow-cytometry-reagents/research-reagents/single-color-antibodies-ruo/559xxx/5598xx/559869_base/pdf/561971.pdf
*https://d1spbj2x7qk4bg.cloudfront.net/en-gb/products/apc-cyanine7-anti-human-cd45ra-antibody-7056?displayInline=true&filename=APC/Cyanine7%20anti-human%20CD45RA%20Antibody.pdf&leftRightMargin=15&pdf=true&topBottomMargin=15&v=20241102123737
*https://d1spbj2x7qk4bg.cloudfront.net/nl-be/products/apc-anti-human-cd45-antibody-705?displayInline=true&filename=APC%20anti-human%20CD45%20Antibody.pdf&leftRightMargin=15&pdf=true&topBottomMargin=15&v=20241102123737
*https://d1spbj2x7qk4bg.cloudfront.net/de-de/products/pe-anti-mouse-cd45-antibody-100?displayInline=true&filename=PE%20anti-mouse%20CD45%20Antibody.pdf&leftRightMargin=15&pdf=true&topBottomMargin=15&v=20241002064001
*https://www.bdbiosciences.com/content/dam/bdb/products/global/reagents/flow-cytometry-reagents/research-reagents/single-color-antibodies-ruo/560xxx/5607xx/560727_base/pdf/560727.pdf
*https://www.thermofisher.com/order/genome-database/dataSheetPdf?producttype=antibody&productsubtype=antibody_primary&productId=15-0339-42&version=Local
*https://www.bdbiosciences.com/content/dam/bdb/products/global/reagents/flow-cytometry-reagents/research-reagents/single-

color-antibodies-ruo/563xxx/5632xx/563217_base/pdf/563217.pdf
*https://d1spbj2x7qk4bg.cloudfront.net/nl-be/products/apc-cyanine7-anti-human-cd235a-glycophorin-a-antibody-14314?displayInline=true&filename=APC/Cyanine7%20anti-human%20CD235a%20(Glycophorin%20A)%20Antibody.pdf&leftRightMargin=15&pdf=true&topBottomMargin=15&v=20241102123737
*https://d1spbj2x7qk4bg.cloudfront.net/en-ie/products/brilliant-violet-421-anti-human-cd71-antibody-15907?displayInline=true&filename=Brilliant%20Violet%20421%E2%84%A2%20anti-human%20CD71%20Antibody.pdf&leftRightMargin=15&pdf=true&topBottomMargin=15&v=20241002064001
*https://www.bdbiosciences.com/content/dam/bdb/products/global/reagents/flow-cytometry-reagents/research-reagents/single-color-antibodies-ruo/560xxx/5600xx/560041_base/pdf/560041.pdf
*https://www.cellsignal.com/products/primary-antibodies/gpx4-antibody/52455?srsltid=AfmBOooMUSBFFTcZ7d42wZjgWnGeimbIuw7RLiZDM9K3zJHv-RdsWBlG
*https://datasheets.scbt.com/sc-8432.pdf

Animals and other research organisms

Policy information about studies involving animals; ARRIVE guidelines recommended for reporting animal research, and Sex and Gender in Research

Laboratory animals	NBSGW female and male mice (7-10 weeks of age) were obtained from Jackson Laboratory (JAX#026622). Mice were housed in a specific pathogen–free facility under controlled conditions (12-hour light/dark cycle, ambient temperature 20–24°C, relative humidity 40–60%) with ad libitum access to autoclaved food and water.
Wild animals	This study did not involve wild animals.
Reporting on sex	Immunodeficient female and male mice were randomly associated to treatment or control groups at the time of transplantation to minimize bias. No specific method of randomization was applied, but animals were distributed across groups to ensure comparable sex and age. Data collection and analysis were not performed blind, but all animals were processed and analyzed under identical conditions. For these reasons, no data points were excluded from the analyses.
Field-collected samples	This study did not involve field-collected samples.
Ethics oversight	All experiments and procedures involving animals were performed with the approval of the Boston Children's Hospital Institutional Animal Care and Use Committee (IACUC #00002257)

Note that full information on the approval of the study protocol must also be provided in the manuscript.

Flow Cytometry

Plots

Confirm that:

☒ The axis labels state the marker and fluorochrome used (e.g. CD4-FITC).

☒ The axis scales are clearly visible. Include numbers along axes only for bottom left plot of group (a 'group' is an analysis of identical markers).

☒ All plots are contour plots with outliers or pseudocolor plots.

☒ A numerical value for number of cells or percentage (with statistics) is provided.

Methodology

Sample preparation	The sample preparation and biological source of the cells were described in the Methods section of the manuscript.
Instrument	Immunophenotypic analyses were performed on LSRII or LSRFortessa (BD Pharmingen). Cell sorting was performed on a BD FACS S6 Symphony (BD Biosciences).
Software	Flow Cytometry data were collected using the BD FACSDiva Software and analyzed with FlowJo.
Cell population abundance	The purity of the sorted samples was determined by rerunning with flow cytometry.
Gating strategy	The gating strategies were described in the manuscript and previous publications.

☒ Tick this box to confirm that a figure exemplifying the gating strategy is provided in the Supplementary Information.
