# Supplementary figures and images for: Inhibiting ferroptosis enhances ex vivo expansion of human haematopoietic stem cells
Source: Nat Cell Biol. 2025 Nov 18;27(12):2214–24. doi: 10.1038/s41556-025-01814-7 (PMC12680438; doi:10.1038/s41556-025-01814-7)

# Source data used in Extended Data Figure 2k

**a**

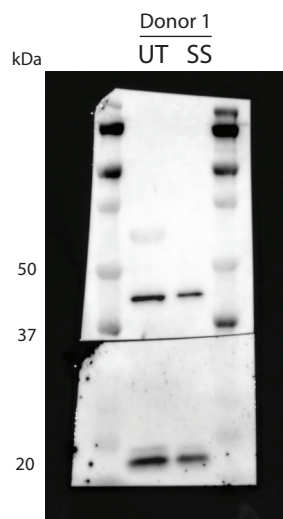

**b**

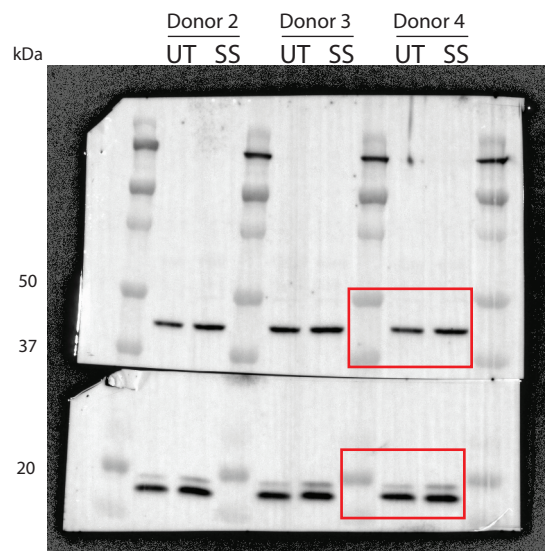

Supplement: Supplementary file 12 — Statistical source data and unprocessed blots of data used in Extended Data Fig. 2k. [file 41556_2025_1814_MOESM12_ESM.pdf]
